# Comparing neural responses to cutaneous heat and pressure pain in healthy participants

**DOI:** 10.1038/s41598-025-99247-7

**Published:** 2025-04-24

**Authors:** Janne Ina Nold, Alexandra Tinnermann, Tahmine Fadai, Marilyn Mintah, Marie-Sophie Morgenroth, Christian Büchel

**Affiliations:** Department of Systems Neuroscience, University Medical Centre Hamburg-Eppendorf, Hamburg, Germany

**Keywords:** Pain, Cutaneous heat, Cuff pressure, fMRI, Nociception, Neuroimaging, Sensory processing, Neuroscience, Neural circuits

## Abstract

Even though acute pain comes in many different shapes and forms, a lot of experimental pain studies predominantly employ cutaneous heat pain. This makes a comparison between different pain types and the link between findings from these experimental studies to clinical pain difficult. To bridge this gap, we investigated both cuff pressure pain and cutaneous heat pain using a within-subject design in combination with functional magnetic resonance imaging (fMRI). Noxious stimuli were applied with a 17-s duration at three different intensities above the pain threshold using a thermode and a computer-controlled cuff pressure device. Both pain modalities led to contralateral activation in the anterior insula and parietal operculum. Heat pain showed greater activation in the precentral gyrus, pontine reticular nucleus, and dorsal posterior insula, whilst pressure pain showed greater activation in the primary somatosensory cortex and bilateral superior parietal lobules. Most importantly, the time course of the fMRI signal changes differed between modalities, with pressure pain peaking in the first stimulus half, whereas heat pain led to a prolonged and increasing response across the stimulus duration with a peak in the second stimulus half. Our findings suggest that pressure and heat pain lead to common as well as different (temporal) activation patterns in key pain processing regions.

## Introduction

Pain can come in many different shapes and forms. To gain a more nuanced understanding of acute pain perception and modulation and their clinical implications it is vital to compare different pain modalities used in experimental studies—such as heat and pressure pain.

To induce pressure pain, some studies^1–4^ have used intramuscular injections of hypertonic saline to induce long-lasting musculoskeletal pain^1^. However, this method is difficult to control over time^5^ and shows substantial inter-individual variability^6^. In contrast, computer-controlled cuff pressure algometry (CPAR) offers a non-invasive and controllable way to induce deep-tissue pain^7–9^, but so far its subcortical and cortical representations have been less explored. One of the few imaging studies^10^ investigating pressure pain using cuff pressure algometry showed intensity-independent activation in the anterior insula (antIns), posterior insula (pIns), secondary somatosensory cortex (S2), and premotor areas. This is complemented by changes in functional brain connectivity between those regions and primary sensorimotor regions in response to a sustained pain state^11^.

In contrast to cuff pressure pain, the neural representations of cutaneous heat pain have received considerably more attention such as in the development of neural signatures of pain^12^ and investigating cerebral contributions to pain beyond nociception^13^. Furthermore, a previous study^14^ in monkeys suggested that noxious heat elicits specific cortical representation within the dorsal posterior insula (dpIns). This proposed regional specificity was also identified in recent human studies employing heat pain, highlighting the parietal operculum (PO)^15^ and dpIns^16^ as regions preferentially encoding painful stimuli as well as pain intensity. Furthermore, activation in the left supplementary motor area (SMA) and the right insula was observed in response to painful input whereas tactile input activated the postcentral gyrus^17^. Further evidence confirms the cortical activation of the posterior insula and adjacent medial operculum in response to heat pain^18^.

Despite the extensive body of research comparing and synthesising neural activation of various pain modalities^19^, a direct comparison of the neural representations underlying cutaneous heat pain and cuff pressure pain has yet to be conducted. To do so, it is crucial to consider different pain intensities to dissociate common and distinct stimulus–response curves^12,15,20–23^. For cuff pressure pain, the primary somatosensory cortex (S1), the primary motor cortex (M1), and the bilateral insula have been identified to show intensity-dependent activation^10^. For heat pain, the pIns and PO have been identified to encode painful stimulus intensity whereas the antIns appears to encode the summation of pain expectation in heat pain^23,24^. Another study highlights the substantial interaction of subcortical (i.e. periaqueductal grey (PAG)) and spinal (i.e. dorsal horn) and cortical structures involved in the intensity-dependent processing of heat pain by showing that the coupling strength of those structures was correlated with individual pain ratings^25^.

Overall, our study had the objective to directly compare cutaneous heat pain (in the following referred to as heat pain) and cuff pressure pain (in the following referred to as pressure pain) concerning common and dissociable neural representations and temporal activation patterns. Importantly, employing different noxious intensities and prolonged pain stimulation allowed us to compare both modalities concerning their stimulus–response curves and temporal activation patterns^15,20,26^.

## Results

**Fig. 1:**
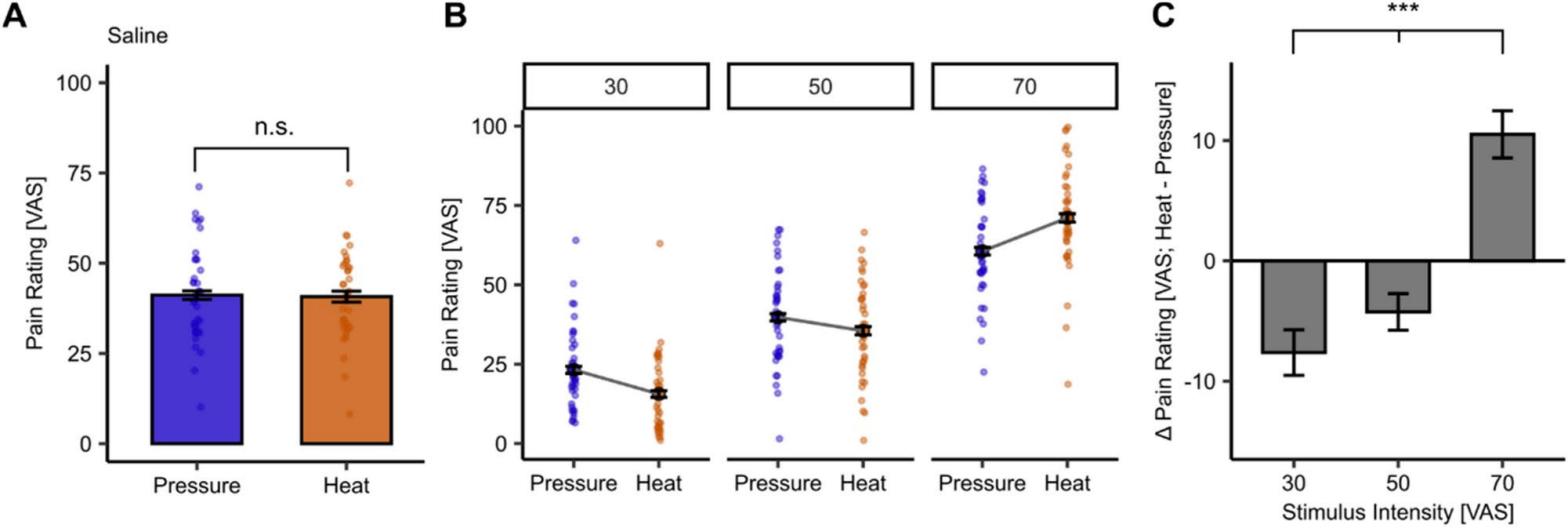
Behavioural results for heat and pressure pain. (**A**) No significant main effect of modality (heat (orange) and pressure (blue)) on pain ratings averaged across all intensities (*p* = 0.78). Bar plots depict average pain ratings with dots showing subject-specific mean ratings across all stimulus intensities. (**B**) Main effect of stimulus intensity on heat pain ratings (*p* < 2 × 10^−16^) and pressure pain ratings (*p* < 2 × 10^−16^). The interaction of stimulus intensity and modality was significant (*p* = 7.25 × 10^−16^). Individual dots depict subject-specific mean ratings at each stimulus intensity (VAS 30, 50, 70) and lines connect mean pain ratings across subjects at each stimulus intensity (VAS 30, 50, 70) in heat and pressure pain. Error bars depict the standard error of the mean (SEM). (**C**) Main effect of stimulus intensity (*p* = 1.44 × 10^−9^) on the difference rating between heat and pressure pain ratings at each stimulus intensity [Heat–Pressure pain rating]. Error bars depict the SEM. n.s. = not significant, *p < 0.05, **p < 0.01, ***p < 0.001.

In the first step, we compared the overall pain ratings for both modalities. There was no significant overall difference in perceived painfulness between heat and pressure pain ratings across all intensities (*β* = − 0.39, SE = 1.38, *t*(1346.56) = − 0.29, *p* = 0.78, Fig. 1A and Supplemental Table S1). Furthermore, both modalities showed a significant effect for increasing stimulus intensity (heat: *β* = 27.78, SE = 0.78, *t*(660.33) = 35.72, *p* < 2 × 10^−16^, Fig. 1B and Supplemental Table S2; pressure: *β* = 0.93, SE = 0.04, *t*(660.69) = 26.62, *p* < 2 × 10^−16^, Fig. 1B and Supplemental Table S3). The corresponding temperatures (in °C) and pressures (in kPa) applied at each VAS intensity are reported in Supplemental Table S4 and the post-hoc paired samples *t*-test calculated between the stimulus intensities for each modality can be found in Supplemental Table S5. Additionally, we observed a significant interaction between stimulus intensity and modality (*β* = 0.45, SE = 0.06, *t*(1359.75) = 8.17, *p* = 7.25 × 10^−16^; Supplemental Table S6), where lower intensity pressure stimuli are perceived as more painful than lower intensity heat stimuli (VAS 30, 50). This pattern was reversed at the highest stimulus intensity (VAS 70) where heat pain was perceived as more painful than pressure pain (Fig. 1C and Supplemental Table S7). Post-hoc paired samples *t*-tests to evaluate the difference between the pain modalities at each stimulus intensity further support this pattern (Supplemental Table S8). Importantly, participants’ pain ratings showed neither sensitisation nor habituation over pain trials across blocks at each stimulus intensity (Supplemental Fig. S1 and Supplemental Table S9). To evaluate potential perceptual differences throughout the stimulus duration in both modalities, we conducted a small behavioural follow-up study with the same modalities in ten different participants using online ratings (for participant characteristics of the follow-up-sample see Supplemental Table S10). The online ratings showed that pressure pain ratings reached a plateau shortly after stimulus onset whereas heat pain ratings increased in painfulness throughout the stimulus duration—especially at VAS 70 (Supplemental Fig. S2).

### Common and distinct neural activation patterns for heat and pressure pain

**Fig. 2:**
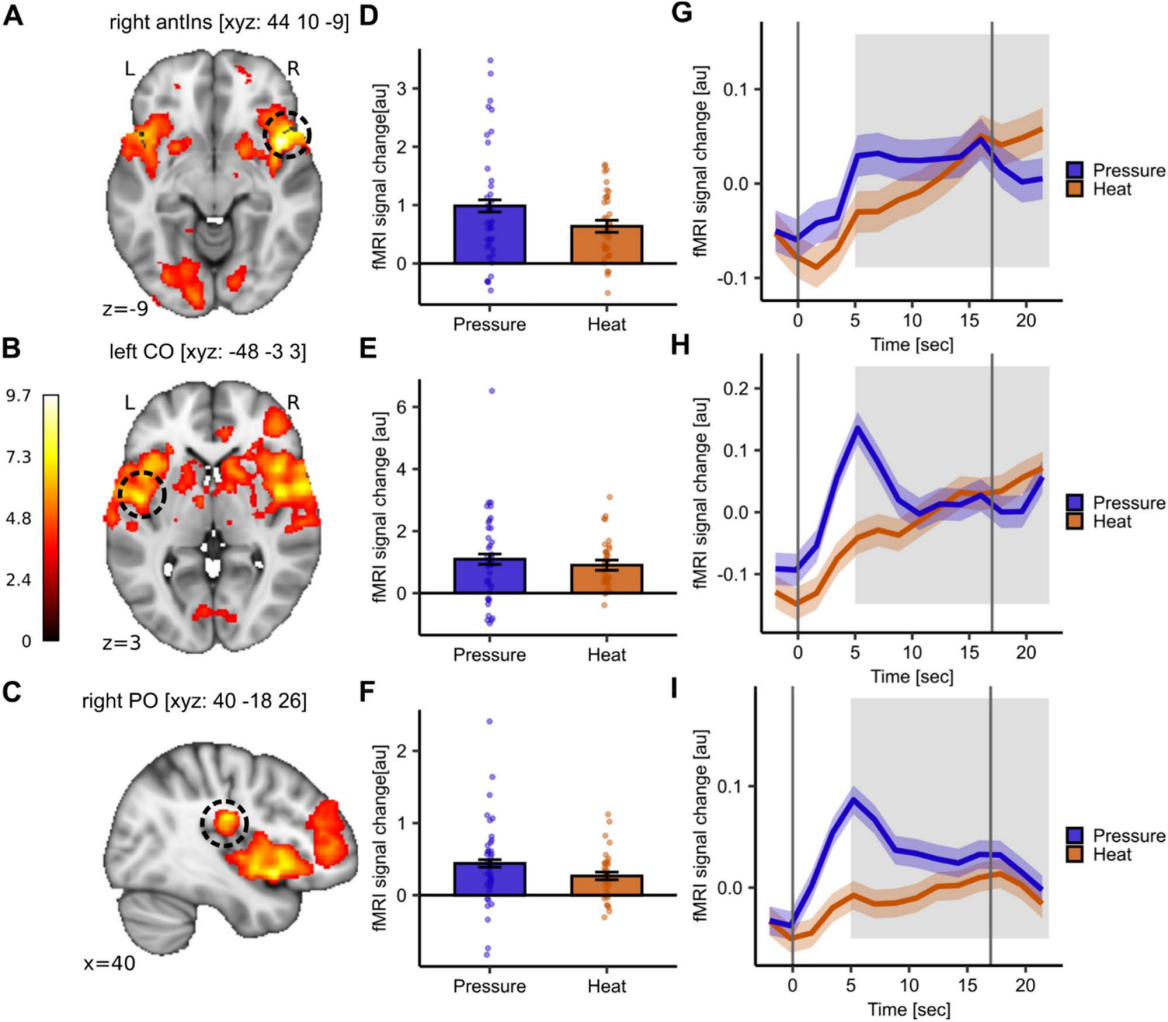
Common neural activation of heat and pressure pain stimulation. (**A**–**C**) Significant common activation patterns from the conjunction analysis of contrasts heat and pressure pain in right anterior Insula (antIns; MNI_xyz_: 44, 10, − 9; T = 9.68, *p*_*WB-FWE*_ < 0.001), left central operculum (CO; MNI_xyz_: − 48, − 3, 3; T = 7.62, *p*_*WB-FWE*_ < 0.001), and right parietal operculum (PO; MNI_xyz_: 40, − 18, 26; T = 7.47, *p*_*WB-FWE*_ < 0.001). (**D**–**F**) Barplots depict mean parameter estimates from boxcar function (i.e. average of stimulus duration) extracted from respective peak voxels and dots show subject-specific mean parameter estimates for heat (orange) and pressure (blue) pain. Error bars depict the SEM. (**G**–**I**) Time course of BOLD response for heat (orange) and pressure (blue) stimuli in the respective peak voxel averaged across all stimulus intensities. The shaded areas around the curves represent the SEM. The grey solid lines indicate stimulus start and stimulus end and the shaded grey area displays the approximate time window for BOLD response (5 s after stimulus onset and stimulus offset). For visualisation purposes, the uncorrected activation maps are displayed at *p*_*uncorr*_ < 0.001.

To identify whether there are common brain regions involved in the perception of both modalities, we first investigated responses across all stimulus intensities. The conjunction analysis revealed a common activation pattern in the right antIns (MNI_xyz_: 44, 10, − 9; T = 9.68, *p*_*WB-FWE*_ < 0.001; Fig. 2A), left central operculum (CO; MNI_xyz_: − 48, − 3, 3; T = 7.62, *p*_*WB-FWE*_ < 0.001; Fig. 2B), and right parietal operculum (PO; MNI_xyz_: 40, − 18, 26; T = 7.47, *p*_*WB-FWE*_ < 0.001; Fig. 2C). In line with the behavioural results, the parameter estimates extracted from the respective peak voxels showed a similar activation pattern for both modalities (Fig. 2D–F). To gain a deeper insight, we extracted the averaged BOLD response time course using a Finite Impulse Response (FIR) model for both modalities in the respective peak voxels. The BOLD time courses of pressure and heat pain differed in the antIns (Fig. 2G) since pressure pain showed a peak in the first stimulus half with a subsequent plateau whereas heat pain increased over the stimulus duration with a peak in the second stimulus half. In the left CO (Fig. 2H) and right PO (Fig. 2I) pressure pain showed a peak in the first stimulus half with a subsequent decline and heat pain showed an increase with a peak in the second stimulus half. The uncorrected activation maps for the individual main effects of heat and pressure pain as well as their conjunction are visualised at *p*_*uncorr*_ < 0.001 in Supplemental Figs. S3–S5.

**Fig. 3:**
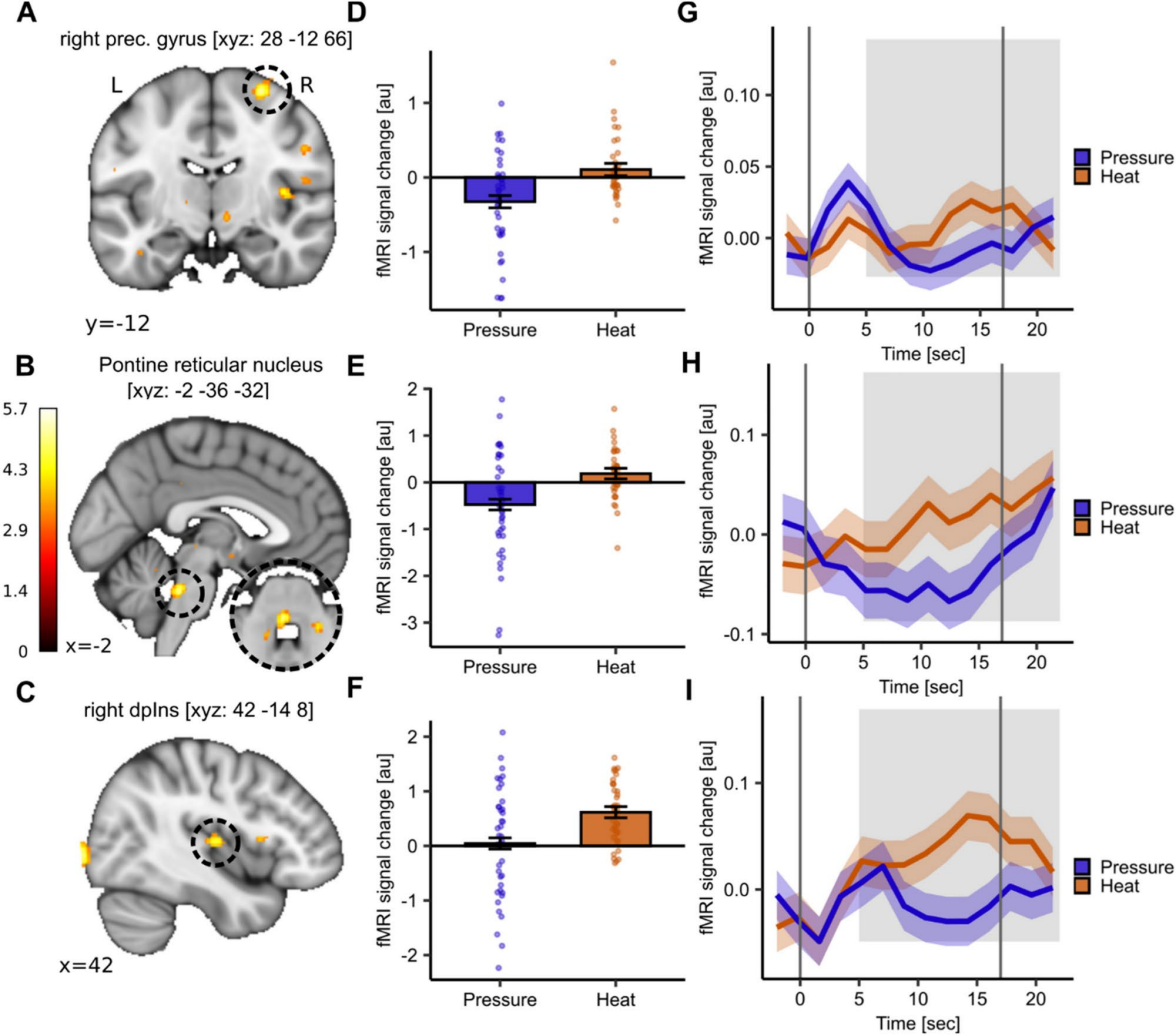
Distinct neural activation of heat pain compared to pressure pain. (**A**–**C**) Significant activation for the contrast heat > pressure pain in right precentral gyrus (MNI_xyz_: 28, − 12, 66; T = 5.19, *p*_*WB-FWE*_ = 0.02) and pontine reticular nucleus (caudal part; MNI_xyz_: − 2, − 36, − 32; T = 5.07, *p*_*WB-FWE*_ = 0.03) as well as approaching significance in the right dorsal posterior insula (dpIns; MNI_xyz:_ 42, − 14, 8; T = 4.73, *p*_*WB-FWE*_ = 0.11). (**D**–**F**) Barplots depict mean parameter estimates from boxcar function (i.e. average of stimulus duration) extracted from respective peak voxels and dots show subject-specific mean parameter estimates for heat (orange) and pressure (blue) pain. Error bars depict the SEM. (**G**–**I**) Time course of BOLD response for heat (orange) and pressure (blue) stimuli in the respective peak voxel averaged across all stimulus intensities. The shaded areas around the curves represent the SEM. The grey solid lines indicate stimulus start and stimulus end and the shaded grey area displays the approximate time window for BOLD response (5 s after stimulus onset and stimulus offset). For visualisation purposes, the uncorrected activation maps are displayed at *p*_*uncorr*_ < 0.001.

As a next step, we investigated whether heat and pressure pain show distinct brain activation patterns. For the contrast heat > pressure pain, we identified the right precentral gyrus (MNI_xyz_: 28, − 12, 66; T = 5.19, *p*_*WB-FWE*_ = 0.02; Fig. 3A) and pontine reticular nucleus (caudal part; MNI_xyz_: − 2, − 36, − 32; T = 5.07, *p*_*WB-FWE*_ = 0.03; Fig. 3B) to show significantly higher activation following heat compared to pressure pain. Despite activation in the right dorsal posterior insula not reaching significance but rather showing a trend (dpIns; MNI_xyz_: 42, − 14, 8; T = 4.73, *p*_*WB-FWE*_ = 0.11; Fig. 3C) it shall be reported for completeness as this region has been shown to be crucial in pain processing^15,16^. The extracted parameter estimates confirmed the higher activation between the modalities in the respective peak voxels averaged across all stimulus intensities (Fig. 3D–F). To gain a better understanding of the stimulus progression over time we extracted the averaged BOLD time course from these peak voxels. Upon visual inspection, heat pain revealed an increase of the BOLD response across the stimulus duration with a peak in the second stimulus half (‘late’ pain) in all peak voxels (Fig. 3G–I, red curve) whereas BOLD activation for pressure pain peaked in the first stimulus half (‘early’ pain) and consequently returned towards baseline (Fig. 3G–I, blue curve). The uncorrected activation map is visualised at *p*_*uncorr*_ < 0.001in Supplemental Fig. S6.

**Fig. 4:**
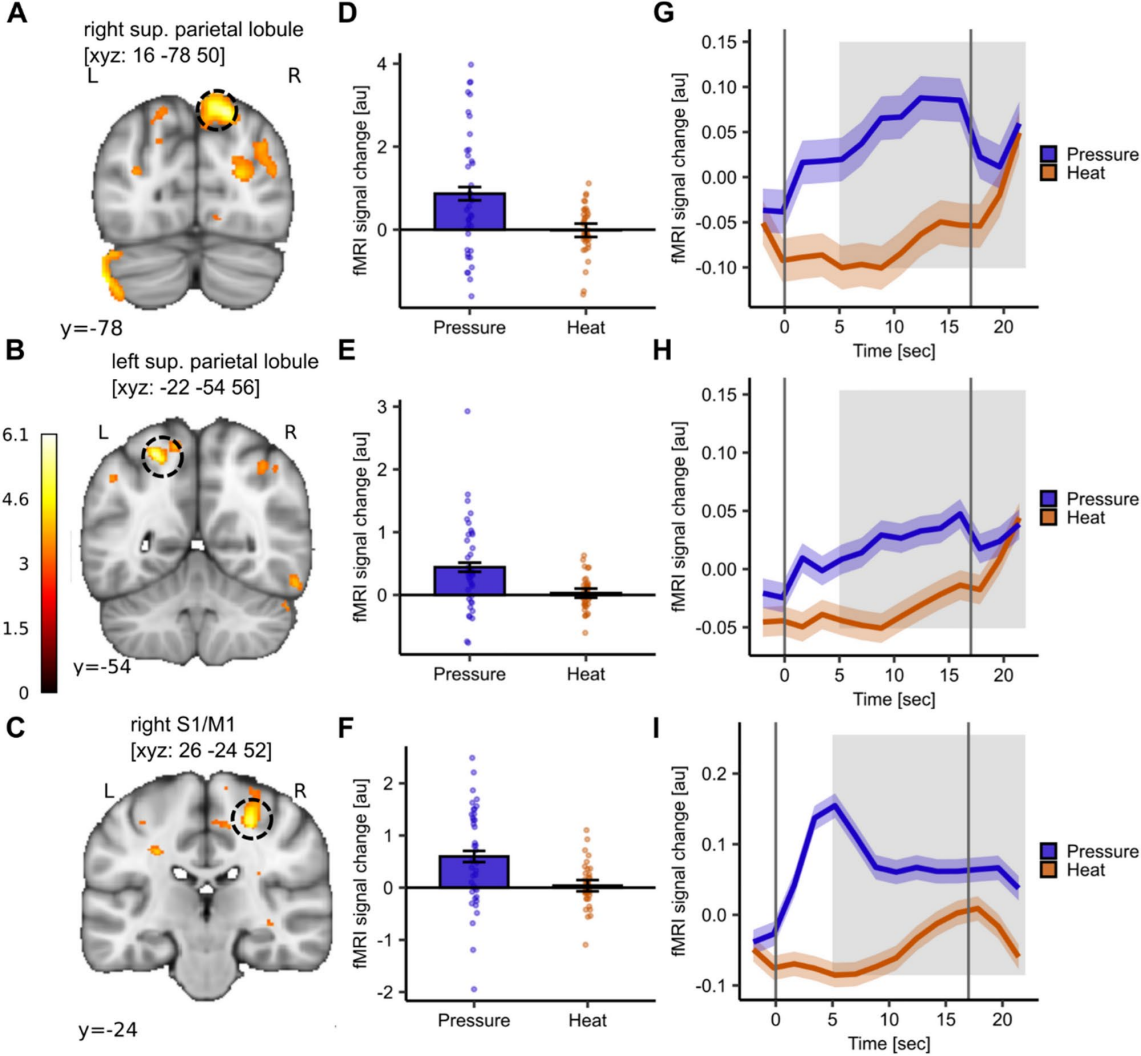
Distinct neural activation of pressure pain compared to heat pain. (**A**–**C**) Significant activation for the contrast pressure > heat pain in the (**A**) right superior parietal lobule (MNI_xyz_: 16, − 78, 50; T = 5.66, *p*_*WB-FWE*_ = 0.002), (**B**) left superior parietal lobule MNI_xyz_: − 22, − 54, 56; T = 5.65, *p*_*WB-FWE*_ = 0.003) and (**C**) right S1/M1 (MNI_xyz_: 26, − 24, 52; T = 5.25, *p*_*WB-FWE*_ = 0.01). (**D**–**F**) Barplots depict mean parameter estimates from boxcar function (i.e. average of stimulus duration) extracted from respective peak voxels and dots show subject-specific mean parameter estimates for heat (orange) and pressure (blue) pain. Error bars depict the SEM. (**G**–**I**) Time course of BOLD response for heat (orange) and pressure (blue) stimuli in the respective peak voxel averaged across all stimulus intensities. The shaded areas around the curves represent the SEM. The grey solid lines indicate stimulus start and stimulus end and the shaded grey area displays the approximate time window for BOLD response (5 s after stimulus onset and stimulus offset). For visualisation purposes, the uncorrected activation maps are displayed at *p*_*uncorr*_ < 0.001.

For the contrast pressure > heat pain, the bilateral superior parietal lobules (right: MNI_xyz_: 16, − 78, 50; T = 5.66, *p*_*WB-FWE*_ = 0.002; Fig. 4A; left: MNI_xyz_: − 22, − 54, 56; T = 5.65, *p*_*WB-FWE*_ = 0.003; Fig. 4B) showed higher activation for pressure compared to heat pain as did the right S1/M1 (MNI_xyz_: 26, − 24, 52; T = 5.25, *p*_*WB-FWE*_ = 0.01; Fig. 4C). The extracted parameter estimates confirmed the significant difference in activation between the modalities in the respective peak voxels (Fig. 4D–F). Again, we extracted the averaged BOLD time course to gain a better understanding of both stimuli and their progression throughout the stimulus duration (Fig. 4G–I). The BOLD activation pattern of both modalities showed a generally greater activation throughout the stimulus duration for pressure compared to heat pain. In S1/M1 (Fig. 4I) pressure pain (blue line) shows a peak in activation during ‘early’ pain followed by a slight decrease in the activation and a steady activation state at a lower level thereafter, whereas heat peaks during the ‘late’ pain phase. The uncorrected activation map is visualised at *p*_*uncorr*_ < 0.001 in Supplemental Fig. S7.

### Temporal activation patterns in heat and pressure pain

**Fig. 5:**
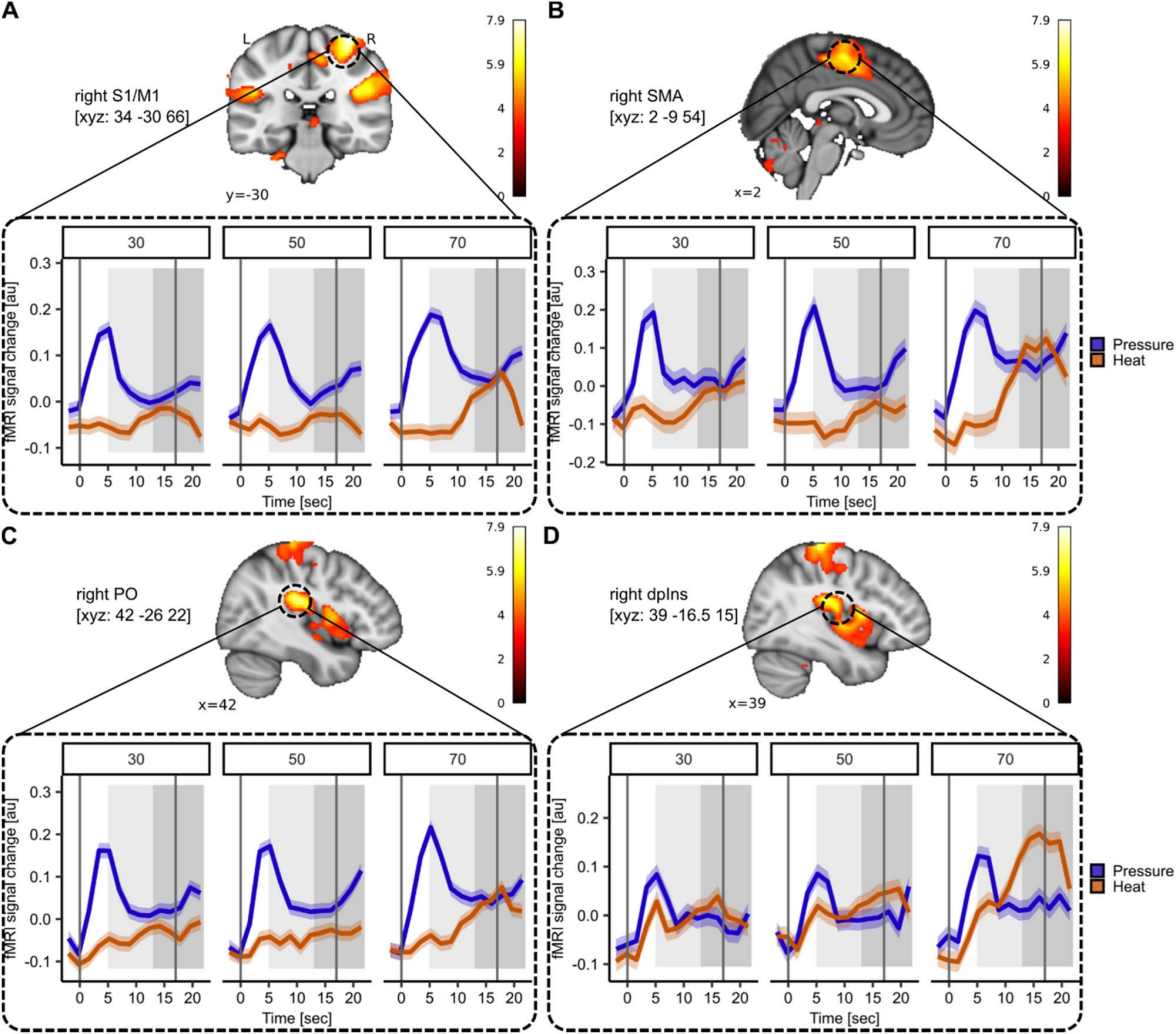
Different neural activation patterns for the ‘early’ and ‘late’ phases in heat and pressure pain. (**A**–**C**) Significant activation for the contrast interaction modality and stimulus half calculated across stimulus intensities in the (**A**) right S1/M1 (MNI_xyz_: 34, − 30, 66; T = 7.91, *p*_*WB-FWE*_ < 0.001), (**B**) right supplementary motor area (SMA; MNI_xyz_: 2, − 9, 54; T = 6.50, *p*_*WB-FWE*_ < 0.001) and (**C**) right parietal operculum (PO; MNI_xyz_: 42, − 26, 22; T = 7.23, *p*_*WB-FWE*_ < 0.001). (**D**) Activation in the right dorsal posterior insula (dpIns) has been visualised for completeness (MNI_xyz_: 39, − 16.5, 15; T = 5.73). The BOLD response time courses for heat (orange) and pressure (blue) stimuli in the respective peak voxels were visualised for each stimulus intensity (VAS 30, 50, 70) for heat (orange) and pressure (blue) pain. The shaded areas around the curves represent the SEM. The grey solid lines indicate the stimulus start and stimulus end. The shaded light grey area displays the approximate time window for BOLD response in the first stimulus half (5 s after stimulus onset) and the shaded dark grey area displays the approximate time window for BOLD response in the second stimulus half (5 s after the first stimulus half end). For visualisation purposes, the uncorrected activation maps are displayed at *p*_*uncorr*_ < 0.001.

As it has become evident from our analyses, heat pain seems to be more pronounced during the ‘late’ pain phase whereas pressure pain is more pronounced during the ‘early’ pain phase. To further investigate this, we statistically compared both stimulus halves for each modality by estimating the interaction of modality and stimulus half across stimulus intensities. The interaction of modality and stimulus half revealed significant activation in the right S1/M1 (MNI_xyz_: 34, − 30, 66; T = 7.91, *p*_*WB-FWE*_ < 0.001; Fig. 5A) and right supplementary motor area (SMA; MNI_xyz_: 2, − 9, 54; T = 6.50, *p*_*WB-FWE*_ < 0.001; Fig. 5B). Furthermore, the right PO (MNI_xyz_: 42, − 26, 22; T = 7.23, *p*_*WB-FWE*_ < 0.001; Fig. 5C) adjacent to the dpIns (MNI_xyz_: 39, − 16.5, 15; T = 5.73; Fig. 5D) showed significant differences for both modalities depending on the stimulus half. The extracted BOLD time course at all stimulus intensities suggests those regions reflect afferent input during different stimulus phases with significantly higher activation in those regions during the ‘early’ phase for pressure pain and the ‘late’ phase for heat pain. This is particularly evident at the highest stimulus intensity (VAS 70). The activation patterns for the individual contrasts heat ‘late’ > ‘early’ and ‘early’ > ‘late’ as well as pressure ‘early’ > ‘late’ and ‘late’ > ‘early’ are visualised in Supplemental Figs. S8–S11.

Interestingly, the behavioural online ratings for heat pain at the highest stimulus intensity (VAS 70; Supplemental Fig. S2, right panel, red line) mirrored the neural response curve shown for heat pain in the PO and dpIns (Fig. 5C,D, right panel, red line). For pressure pain, however, the behavioural online ratings at the highest stimulus intensity (Supplemental Fig. S2, right panel, blue line) matched the neural response curve identified in the right antIns (Fig. 2G, blue line) since it reaches a plateau for the stimulus duration.

As a next step, we investigated the stimulus–response mapping of the three stimulus intensities for both pain modalities. The conjunction analyses showed no significant overlap between heat and pressure in their intensity encoding (Supplemental Fig. S12) and neither did the interaction of modality and stimulus intensity (Supplemental Figs. S13 and S14). The parametric contrasts for heat and pressure pain individually are shown in the Supplemental Figs. S15 and S16, respectively. We have furthermore reported the contrasts for the difference and conjunction between heat and pressure pain at each stimulus intensity in the Supplemental Figs. S17–S25. Due to the fact that heat and pressure pain showed different temporal dynamics, we also investigated the parametric contrasts in both stimulus halves for both modalities. The activation patterns for parametric contrast for ‘early’ pressure pain and ‘late’ heat pain did not deviate from the parametric contrasts spanning the complete stimulus duration.

### Neurologic Pain Signature (NPS) response to heat and pressure pain

**Fig. 6:**
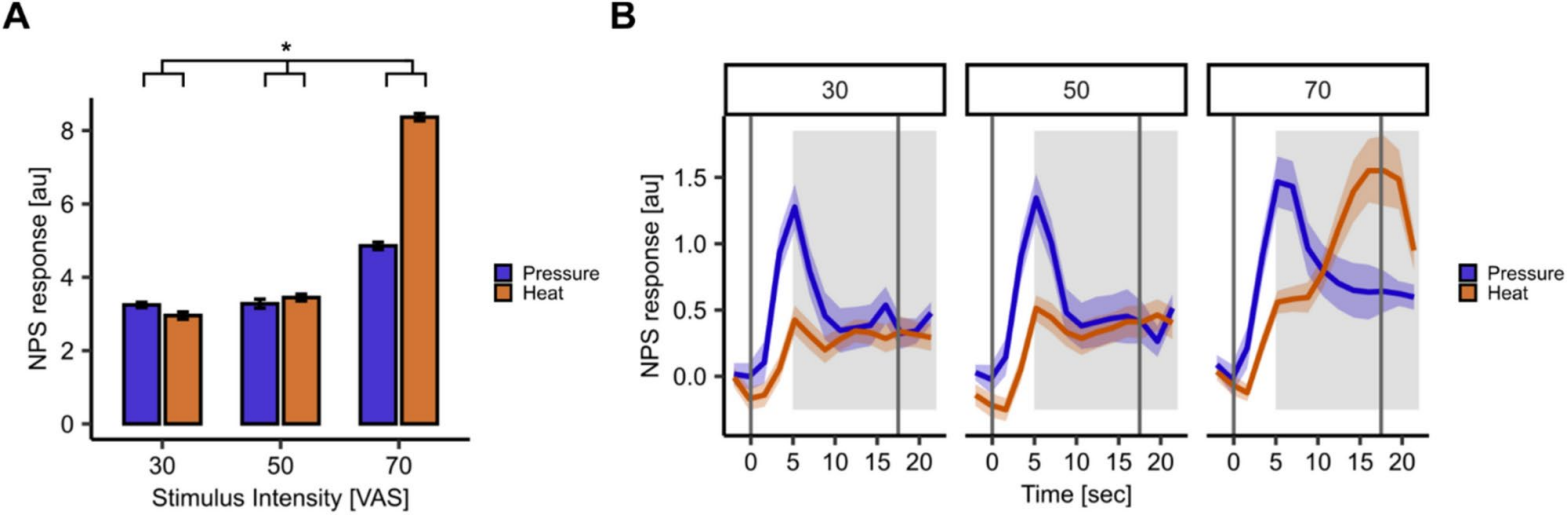
Neurologic Pain Signature (NPS) response. (**A**) NPS scores extracted from normalised and smoothed first-level contrast images of each intensity contrast (VAS 30, 50, 70) and each modality (heat (orange) and pressure (blue)) multiplied with the NPS mask showed a significant interaction of modality and stimulus intensity (*p* = 0.02) with the greatest response to heat pain at the highest stimulus intensity (VAS 70). Error bars indicate SEM. (**B**) Time course of NPS estimates shown for both modalities (heat and pressure pain) at all stimulus intensities (VAS 30, 50, 70). Shaded areas around the curves represent SEM. The grey solid lines indicate stimulus start and end and the shaded grey area displays the approximate time window for BOLD response (5 s after stimulus onset).

Additionally, we evaluated whether the neural response patterns identified in individual brain regions were mirrored across several brain regions involved in pain perception by using the NPS^12^. The NPS assigns different weights to different voxels depending on their contribution and correlation to pain perception^12^. The resulting NPS scores of each modality and intensity showed a significant interaction of stimulus intensity and modality on NPS scores (*β* = 0.09, SE = 0.04, *t*(192) = 2.27, *p* = 0.02, Fig. 6A and Supplemental Table S11). Since the neural response curves also differed between modalities and brain regions depending on the pain phase (‘early’ or ‘late’ pain) we estimated the NPS time course at every repetition time (TR) across the NPS at each stimulus intensity (Fig. 6B).

**Fig. 7:**
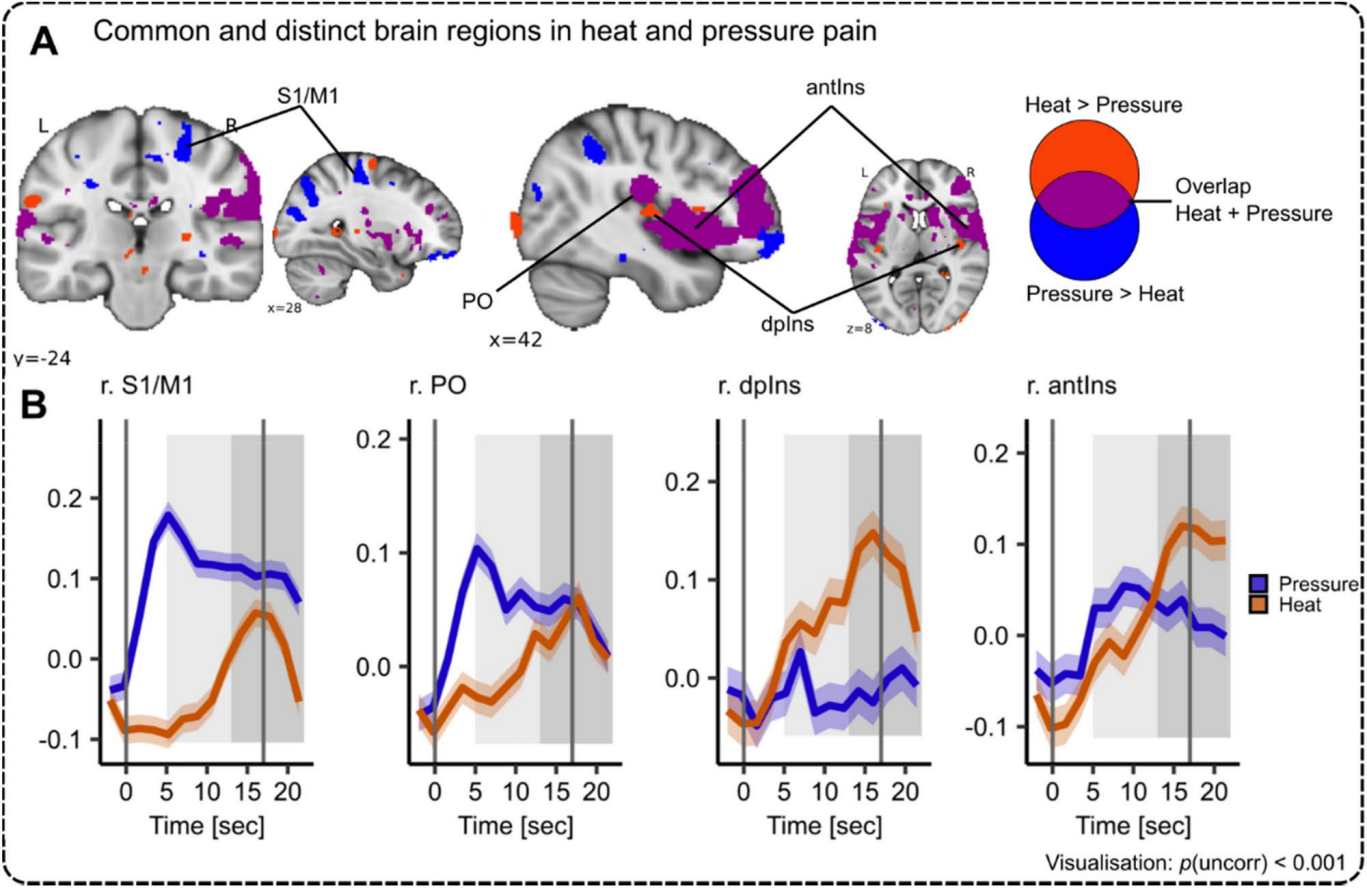
Overview of neural and temporal activation patterns in response to heat and pressure pain. (**A**) Common (purple: conjunction heat and pressure pain) and distinct (red: heat > pressure pain; blue: pressure > heat pain) cortical activation in response to heat and pressure pain (*p*_*FWE-WB*_ < 0.05). (**B**) Exemplary response curves for heat (orange) and pressure (blue) time course extracted from respective significant peak voxels at highest stimulus intensity (VAS 70). Shaded areas around the curves represent SEM. The grey solid lines indicate the stimulus start and end. The shaded light grey area displays the approximate time window for BOLD response in the first stimulus half (5 s after stimulus onset) and the shaded dark grey area displays the approximate time window for BOLD response in the second stimulus half (5 s after the first stimulus half end). n.s. = not significant, *p < 0.05, **p < 0.01, ***p < 0.001.

Finally, we visualized commonalities and differences between heat and pressure (Fig. 7A) and depicted areas with the highest differences in time courses (Fig. 7B). Intriguingly, the temporal activation pattern in the NPS at the highest stimulus intensity is mirrored in the temporal activation pattern at the highest stimulus intensity in common brain regions such as the PO and antIns (Fig. 7A,B). Furthermore, the neural response curve for pressure pain showed a peak during ‘early’ pain (Fig. 7B, blue line), whereas heat pain during ‘late’ pain (Fig. 7B, orange line) across the NPS – especially at the highest stimulus intensity (Fig. 7B, right panel).

## Discussion

In this study, we investigated behavioural responses and brain activation patterns elicited by acute cutaneous heat pain and cuff pressure pain. Although heat and pressure pain were on average perceived as similarly painful, we identified a significant interaction of stimulus intensity and modality, where pressure pain was perceived as more painful at lower intensities and heat pain was perceived as more painful at the highest stimulus intensity. In the brain, we identified overlapping activations for both modalities in the anterior insula (antIns) and parietal operculum (PO) contralateral to the stimulation site. Furthermore, heat pain showed higher activation in the right precentral gyrus, pontine reticular nucleus, and right dorsal posterior Insula (dpIns). In contrast, pressure pain showed higher activation in the right primary somatosensory and motor cortex (S1/M1) and bilateral superior parietal lobules. The temporal dynamics of both modalities differed in key pain processing regions such as S1/M1, supplementary motor area (SMA), PO, and adjacent dpIns with heat pain showing an increase in activation throughout the stimulus duration with a peak during ‘late’ pain whereas pressure pain showed a peak during ‘early’ pain. Overall, our findings suggest common and distinct neural activation patterns for heat and pressure pain that are characterised by different temporal activation patterns.

Our behavioural results show that, on average, heat and pressure pain are perceived as similarly painful. Upon closer inspection, there is a difference in perceived painfulness at the highest stimulus intensity with heat pain showing higher pain ratings. This might be due to different perceptual qualities of heat and pressure pain; the latter potentially being perceived as uncomfortable rather than painful. Furthermore, due to the prolonged compression by the cuff and the resulting blood flow restriction the pressure stimulus could cause an additional feeling of discomfort.

### Common and distinct neural representations of heat and pressure pain

We identified common activation in the antIns and PO. Activation in the antIns has been shown to reflect salience and attentional processes^27–30^ as well as negative affect^31^. Furthermore, the antIns also encodes pain expectation and prediction errors^23^, the latter being correlated with increased salience^15^. Studies investigating cuff-induced pressure pain^10,32^ also reported activation of the antIns. The PO as part of S2 has been established to be crucial in pain processing^33,34^. In a previous study^15^, the PO showed activation in response to heat pain but not salience, suggesting a distinct role in pain processing. Our results further confirm the activation of the PO in pressure pain processing^10^. The overlapping activation in those key structures relevant to the encoding of salience and attentional processes indicate common circuits to be employed in heat and pressure pain.

In terms of distinct activation patterns, heat pain showed significantly higher activation in the right precentral gyrus and pontine reticular nucleus, as well as activation in the right dpIns approaching significance compared to pressure pain. The precentral gyrus is part of the motor system and has been reported to show activation in response to motor tasks^35^. In the current study, the reported peak activation in the precentral gyrus is located in the dorsal premotor cortex (PMd)^36^ that is associated with motor planning or preparation^37^ and a more general sensorimotor integration^38^. Interestingly, the FIR model extracted from the peak voxel revealed that heat and pressure pain both show an initial peak after the stimulus onset but heat pain showed an additional second peak towards the end of the stimulus (Fig. 2G). One potential explanation might be that due to the prolonged stimulus duration, heat pain might elicit stronger behavioural responses (i.e. the urge to withdraw from a harmful stimulus). However, it remains unclear why activation in the PMd is stronger in heat compared to pressure pain and warrants further investigation.

The activation peak in the brainstem is located in the pontine reticular nucleus which is part of the reticular formation and has been extensively studied in the context of top-down and bottom-up pain modulation^39^. This region has been shown to receive bilateral projections from spinal cord afferent projecting nociceptive inputs^40–42^ predominantly originating from the dorsal horn^43,44^ and is highly relevant for modulating pain perception in a top-down and bottom-up manner. Previous research^45,46^ using electrophysiological in vivo recordings in the rat has shown that this region contains neurons that are exclusively activated by noxious stimuli activating Aδ and C-fibres (i.e. cutaneous heat pain). Since our findings showed greater activation of the pontine reticular nucleus in heat compared to pressure pain this might be due to the specialised nociceptive processing of stimuli conveyed by Aδ and C-fibers.

Furthermore, we identified the right dpIns to show stronger activation in heat compared to pressure pain, despite not reaching significance. Previous research in monkeys using tract-tracing has identified sub-regions of the dpIns to receive modality, intensity, and location-dependent nociceptive input^14,47^. In humans, neuroimaging studies have also shown a somatotopic organisation of the dpIns in response to painful heat^48^ and further established the contralateral dpIns to represent discriminative thermal nociceptive input^49^. More recent work has linked the dpIns tightly to pain^16^ including intensity-related activation in response to noxious heat^15^. Our results further suggest that the dpIns might respond stronger to heat pain than to pressure pain.

Of note, we identified greater activation in S1 as well as bilateral superior parietal lobules in pressure pain compared to heat pain. Generally, S1 is part of a larger network involved in pain perception^33^ and has been reported to play a vital role when encoding sensory features of pain^50^ with its activation being closely linked to an individual’s perception of different pain intensities^22^. The activation of S1 in response to pressure pain is in line with previous studies using cuff pressure to induce pain^10,11^. Additionally, connectivity changes in S1 have been shown in response to sustained pain with the sensorimotor network and salience network^11^. In this study, we have used a cuff to induce pressure pain, which stimulates a larger area compared to heat pain and thus might lead to more input to the somatosensory cortex resulting in stronger S1 activation. In addition, S1 plays a vital role in integrating nociceptive input^51^ in processes such as temporal summation^52,53^. For cuff pressure pain, spatial and temporal summation have been identified behaviourally^9,54,55^ but have not yet been validated in the brain. Our results further support the crucial role of S1 in pressure pain.

Furthermore, previous studies report S1 to show an intensity-related BOLD response at lower^26^ and higher^10,22^ stimulus intensities. Despite activation of S1 also having been reported for heat pain^33,56^ our results suggest a greater activation induced by pressure pain.

Our data also show stronger activation in the bilateral superior parietal lobules for pressure compared to heat pain. The superior parietal lobule is involved in perception^57,58^, attention^59^, and higher-order cognitive processes^60,61^. The pressure cuff used to induce pressure pain in the current study covers a large skin area, which might result in increased spatial awareness of the location of the cuff and, thus, might induce stronger activation of the superior parietal lobule as compared to a more focal heat pain stimulus.

### Temporal dynamics in heat and pressure pain

When comparing the temporal activation patterns in both modalities it becomes evident that heat and pressure pain show different peaks in the ‘late’ (second stimulus half) and ‘early’ (first stimulus half) pain phase, respectively. The difference in these temporal activation patterns is evident in key brain processing regions such as the S1, PO, and dpIns. The separation of heat pain into discrete pain phases (‘early’ and ‘late’) has been observed by other studies investigating the role of endogenous opioids^62–64^ or placebo effects^62,65^. Furthermore, early naloxone studies in healthy^66^ and chronic pain populations^67^ suggested the opioidergic effect to be primarily evident in later pain phases when a certain stimulus intensity is reached.

Apart from inducing greater activation in S1, pressure pain peaked during the ‘early’ pain phase followed by a slight decrease in the activation and a steady activation state at a lower level thereafter whereas heat pain showed a peak towards the end of the stimulus duration. In line with these findings, the temporal activation pattern in S1 for pressure pain has been reported to show an onset peak in the first stimulus half with activation remaining on a plateau until the stimulus offset^10^. Since the behavioural online ratings in the follow-up sample also revealed perceptual differences in heat and pressure pain across stimulus duration, one can assume that these perceptual differences are reflected in S1.

Interestingly, our results also convey these differing temporal activation patterns in the right PO and dpIns with pressure showing a peak during ‘early’ and heat during ‘late’ pain. This difference could be driven by several factors. Firstly, the initial peak activation in the PO and dpIns in the ‘early’ pain phase in pressure pain might be an indication that these brain areas are sensitive to changes in perception which are most pronounced at the onset of stimulation. Secondly, this finding could hint at potentially different mechanisms employed by cutaneous heat and cuff pressure pain already at the receptor level. Due to the larger stimulated skin area, theoretically, the cuff pressure can exert more mechanical stress and strain on the superficial and deeper tissue along with exciting more nociceptors^68^. Based on earlier animal studies comparing mechanosensitivity in deep tissue and skin nociceptors^69,70^ it has been proposed that the mechanosensitivity of nociceptors in deep tissue is lower than in superficial tissue^68^. A more recent study in humans has further shown that non-painful cuff pressure is also relayed via mechanoreceptors that are related to faster myelinated Aβ afferents^71^. Another recent study^72^ in humans has further identified thickly myelinated extremely fast-conducting cutaneous mechanoreceptors that respond to painful mechanical pinprick stimulation but not to touch. However, it should be noted that noxious pinprick stimulation might differ from noxious cuff pressure stimulation in the afferent fiber types activated. This, potentially, has important implications for various clinical pain conditions, such as musculoskeletal pain, which frequently originates from deeper tissues^7,73^. In contrast to cuff pressure pain, cutaneous heat pain is relayed via thermoceptive (slower C fibers) and nociceptive fibers (faster Aδ and C fibers). These different fiber types might affect information transmission at an early stage of nociceptive processing and might contribute to the difference in stimulus–response curves in key brain regions of both modalities.

Corroborating this, the temporal dynamics estimated for the NPS at the highest stimulus intensity (Fig. 6B, rightest panel) mirrored the temporal dynamics shown in the right PO (Fig. 7B, middle left panel) and dpIns (Fig. 7B, middle right panel), as well as the antIns (Fig. 7B, right panel) where the neural response curve for pressure pain showed a peak during ‘early’ pain, whereas for heat pain it showed a peak during ‘late’ pain. It must be noted, that the NPS has been established mainly based on studies employing heat pain but not pressure pain^12^, potentially not capturing the neural activation for pressure pain as accurately as for heat pain.

Some limitations apply to this study when interpreting the results. For one, we compared both modalities without further relying on additional measures such as skin conductance response (SCR), which is a reliable measure when comparing different sensory modalities regarding salience^15^. Furthermore, we applied the different pain modalities on the left upper and lower arm to allow for better comparability of lateralised brain activation patterns. Still, activation patterns might differ due to the different positions of stimulation. Nonetheless, given the segmental orientation of spinal segments on the arm this is unlikely as our stimulation supposedly included segments C5 and C6 for heat and pressure pain^74^. Finally, the data presented in this study was collected after the low-intensity exercise control condition. Thus, participants were not completely sedentary before receiving the pain stimuli. However, it is unlikely that this has biased the results since the same exercise condition has been performed before receiving both painful stimuli.

In summary, our results show that pressure and heat pain lead to common as well as distinct spatial and temporal activation patterns in key pain processing regions. This suggests a crucial influence of stimulus duration and intensity and has important implications for understanding pain physiology as well as modulating mechanisms in different pain modalities.

## Methods

This study was part of a larger project investigating exercise-induced pain modulation in heat and pressure pain using a pharmacological intervention. The overall project was preregistered with the WHO-accredited *Deutsches Register Klinischer Studien* (DRKS; ID: DRKS00029064) before conducting the research, adheres to the requirements of the Declarations of Helsinki^75^, and was approved by a local ethics committee (*Ärztekammer Hamburg*). The preregistered hypotheses included the comparison of heat and pressure pain which shall be investigated here. Nevertheless, these analyses serve as an exploratory basis for future investigations involving heat and pressure pain and were not explicitly preregistered.

### Participants

Forty-eight healthy, right-handed participants were invited to take part in the study. Upon entering the study, participants provided informed consent and were screened for anxiety (State-Trait Anxiety Inventory; STAI)^76^, depression (Beck Depression Inventory 2; BDI-II)^77^ as well as potential MR contraindications. Furthermore, participants’ body mass index (BMI) was required to range between 18 and 30. The age of participants was between 18 and 45 years. Eight participants were excluded from the original study for the following reasons: Two participants withdrew from the study due to circumstantial reasons (moving, no appointment found), and one participant was excluded due to misunderstanding the pain calibration procedure (i.e. lower ratings for more painful stimuli and higher ratings for less painful stimuli) and one participant due to a BMI score below 18. Two participants did not wish to continue the study after day two due to personal reasons. Two participants were excluded after day two due to a syncope when administering saline in the original study protocol. Upon data screening, one subject was excluded due to excessive movement in the MR scanner. A total of thirty-nine participants (Age: *M* = 26.2, *SD* = 4.8, 21 female) were included in the final sample (for participant characteristics see Supplemental Table S12).

### Paradigm

**Fig. 8:**
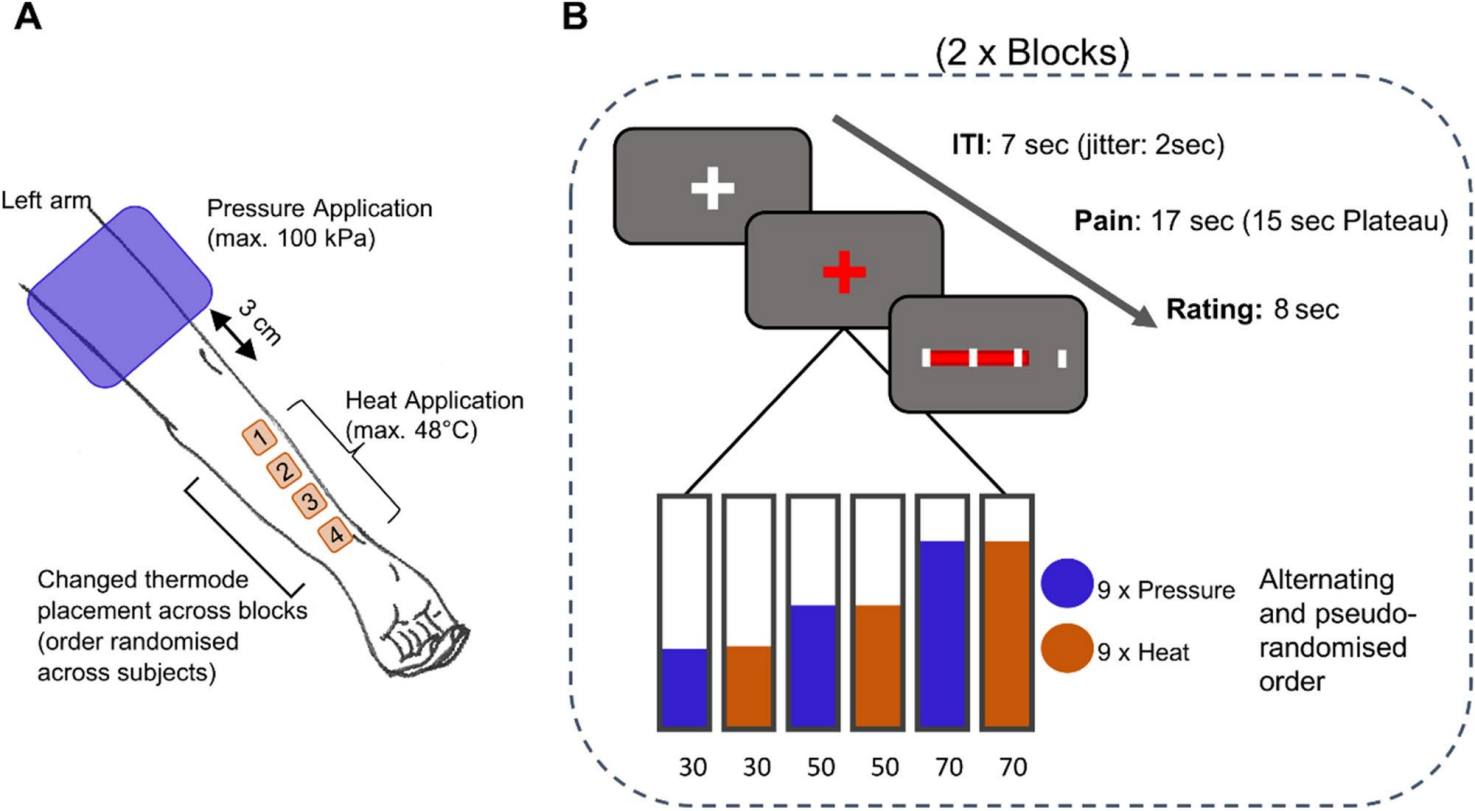
Experimental design. (**A**) Stimulus application of heat pain (max. 48 °C) and cuff pressure pain (max. 100 kPa) on the lower and upper left arm, respectively. (**B**) Two blocks with alternating heat (9×) and pressure (9×) pain at three different intensities (VAS 30, 50, 70). Each stimulus had a 15-s plateau and was followed by an 8-s VAS rating.

The original study consisted of two identical experimental days with the only differences lying in the pharmacological treatment administered (naloxone or saline). Each experimental day consisted of four blocks with each block comprising a 10-min cycling at high or low intensity that was immediately followed by a 15-min fMRI scan (more details on the original experimental paradigm can be found in the Supplemental Materials). For the current study, only blocks after the exercise control condition (low-intensity) and only from the placebo (saline injection) study day were included in the analyses. An initial calibration day took place one week before the MR session, where both modalities were calibrated using an identical procedure resulting in three supra-threshold pain intensities (30, 50, 70 out of 100). On the experimental day, a recalibration inside the MR scanner took place. All stimulus presentation was realized using MATLAB R2016b and Psychophysics Toolbox (Version 3.0.19). Physiological data, including respiration and heart rate, was recorded at 1000 Hz with the Expression System (In Vivo, Gainesville, USA) using the spike2 software and a CED1401 system (Cambridge Electronic Design). Heat stimulation was applied using a thermode (TSA-2, Medoc, Israel) attached to the left lower arm where four 2.5 × 2.5 cm squares were drawn below each other and numbered (Fig. 8A) since the position of the thermode was changed before every block and this order was randomized across participants. Each heat stimulus lasted approximately 17 s in total with a plateau of 15 s and a ramp speed of 13 °C/s. The ramp-up time for heat stimuli until the plateau was reached ranged between 0.8 and 1.2 s (*M* = 1.05, *SD* = 0.09) with a baseline temperature of 32 °C between heat stimuli. For the pressure stimuli, a computer-controlled cuff pressure algometer (CPAR; NociTech, Denmark, and Aalborg University, Denmark) consisting of a compressor tube and 13 cm wide tourniquet cuff (VBM medical, Sulz, Germany, 61 cm length) was used. The cuff was mounted to the left upper arm with a 3 cm distance to the cubital fossa to exert pressure on the upper arm tissue (Fig. 8A)^55^. Additionally, a protective tubular elastic dressing (Tricofix, D/5, 6 cm × 20 cm) was positioned underneath the cuff to protect the bare skin from potential harm. The pressure was applied by inflating the chambers of the cuff with a maximal pressure limit of 100 kilopascals (kPa) and a ramp speed of 30 kPa/s. The stimulus length of the pressure stimuli was also approximately 17 s (ramp-up, 15-s plateau, ramp-down) (Fig. 8B). The ramp-up time for pressure stimuli until the plateau was reached ranged between 0.1 and 3.3 s (*M* = 1.64, *SD* = 0.66) and returned to 0 kPa between pressure stimuli. Each stimulus was accompanied by a red fixation cross as a visual cue. Following each stimulus, the painfulness of the previous stimulus was rated on a Visual Analog Scale (VAS) from 0 (minimally painful) to 100 (almost unbearably painful) by using a button box. Participants were explicitly instructed to rate the maximum perceived pain over the 17-s duration. The rating took place within 8 s, followed by an inter-trial interval (ITI) of 7 s (jittered by ± 2 s) before commencing with the next trial. One MRI block consisted of nine heat and nine pressure stimuli alternating at three intensity levels (30, 50, 70 VAS) in a randomized order (Fig. 8B). Following the task, a field map was acquired after each block. Overall, participants spent four blocks inside the MR scanner with each block lasting 15 min. Of those four blocks, only those after low-intensity exercise were included in the analyses.

In a behavioural follow-up study, ten different participants received painful stimuli outside the MR scanner and rated both heat and pressure stimuli calibrated at 30, 50, and 70 VAS during the whole duration of their administration as well as 2 s after (for participant characteristics see Supplemental Table S10). This study was conducted to investigate the behavioural response to painful stimuli throughout their duration. Thus, the VAS scale was adapted to also include non-painful perception below the pain threshold ranging from 0 (no perception) to 150 (almost unbearably painful) with the pain threshold at 50 (minimally painful). Participants were instructed to rate throughout the stimulus duration corresponding to the perceived painfulness and only rate minimally painful (corresponding to 50) or above if the pain threshold was reached or exceeded. When there was no change in perception participants did not change the VAS rating.

### Behavioural data analyses

Behavioural data analyses were performed using MATLAB 2021b and RStudio (Version 2021.09.1). We conducted Linear Mixed Effect Models (LMER)^78,79^ using the *lmer* function from the *lme4* package (Version 1.1-35.1)^80^ in R. Only data from the placebo study day (saline injection) and exercise control condition were included in the analyses. The pain ratings served as the dependent variable and subject as well as the trial number as random effects in every model. The treatment order (saline or naloxone study day first) was included as fixed effect in every model to account for potential variance. Concerning the difference in pain ratings between heat and pressure pain (across stimulus intensities), modality was included as fixed effect in the LMER model. To investigate the interaction of stimulus intensity and modality on pain ratings, the interaction term has been included as fixed effect in a further LMER model. To further investigate the effect of stimulus intensity on both modalities individually, separate LMERs have been conducted with stimulus intensity as fixed effect and heat or pressure pain ratings as dependent variable. Finally, the difference score between heat and pressure pain ratings [Heat–Pressure ratings] has been calculated and included as the dependent variable in the LMER with stimulus intensity as fixed effect.

For the follow-up study the online ratings of both heat and pressure stimuli were sampled at 70–75 samples/second throughout the stimulus duration and 2 s after the stimulus end. For analysis purposes, the continuous ratings were interpolated at 0.9 s (1/2 of TR 1.8 s).

### MRI data acquisition

The T1-weighted anatomical images (coronal slice orientation) were acquired with a voxel size of 1 × 1 × 1 mm^3^ using a 3D-MPRAGE sequence (TR: 7.1 ms, echo time (TE): 2.98 ms; flip angle: 9°, 240 slices, slice thickness = 1 mm, field of view (FOV) = 256 mm). Functional magnetic resonance imaging (fMRI) data were obtained using a 3 Tesla Siemens system (Magnetom PRISMA; Siemens Healthcare, Erlangen, Germany) equipped with a 64-channel head coil. Sixty slices were recorded with a slice thickness of 2 mm using T2*-weighted echo-planar imaging (EPI) employing a multiband factor of 2 (TR: 1.8 s; TE: 26 ms; flip angle: 70°). The FOV was set at 240 mm, encompassing the upper part of the medulla and the brainstem as well as allowing for whole brain coverage. This resulted in a voxel size of 2 × 2 × 2 mm^3^. Four volumes were discarded at the onset of each run. Shimming and auto-alignment procedures were performed at the outset of each block and before the acquisition of EPI images.

### Preprocessing

The MRI data was analysed using SPM12 (Wellcome Trust Centre for Neuroimaging, London, UK) and MATLAB 2021b (The Mathworks Inc. 2021b). The brain data was processed using a standardized SPM12-based pipeline (https://github.com/ChristianBuechel/spm_bids_pipeline.git) supporting BIDS format^81^ to ensure data accessibility and reproducibility. The T1-weighted anatomical images were acquired on the first experimental day. The functional images were then slice time corrected and realigned using rigid-body motion correction with six degrees of freedom. Following this, a non-linear coregistration^82^ was performed using the mean EPI from the realignment and the T1 image as implemented in the CAT12 SPM toolbox. In short, this approach segments the T1 weighted image and creates a subject-specific T1 weighted template. This template is then used for a non-linear registration of the mean EPI image using the unified segmentation approach as implemented in SPM^83^. Therefore, it aligns the mean EPI and individual anatomical (T1-weighted) image with high spatial accuracy and can produce better subject-specific alignment (as compared to a linear coregistration). This has already been used in previous studies from our group^15,84^. After this, the T1 image was normalized to MNI space (MNI152NLin2009cAsym as provided by CAT12 toolbox) using DARTEL. Subsequently, transformation fields were created by combining the deformation field from the non-linear coregistration with the DARTEL flow fields to map the EPI images to T1 and template space. A mask for the 1^st^-level GLMs was created based on the GM and WM partitions of the T1 image and then warped into EPI space and smoothed with a 3 mm full width at half maximum (FWHM) Gaussian kernel. Then a second realignment took place but constrained by a brain mask to remove the effects of eye movements. Finally, all EPI images were resliced in their individual space. Noise regressors for the first level analysis were created using six principal components of white matter responses and six principal components of CSF responses^85^ as well as a ROI at the posterior tip of the lateral ventricle comprising the WM CSF interface (as used previously^15^). As a final step, careful quality checks took place of all functional images generated after realignment, nonlinear co-registration, and normalization through visual inspection.

### First-level analyses

The single-subject analyses of the brain data were performed in native space using a General linear model (GLM) approach. Two separate models were calculated, with the first model containing the complete stimulus duration and the second containing a split stimulus duration. For both models, the design matrices for each subject comprised both fMRI blocks, each containing the stimulus onsets for heat and pressure stimuli at all three intensity levels (VAS 30, 50, 70), the VAS rating, button presses, and a session constant. For the second model, we additionally split the stimulus duration into two equal parts (~ 8.5 s each) that resulted in ‘early’ (stimulus onset—8.5 s) and ‘late’ pain (8.5 s—stimulus end) regressors for heat and pressure, resulting in 12 regressors. Again, the VAS rating, button presses, and a session constant were also included in the design. Since the VAS pain ratings for pressure pain started while the stimulus ramped down and not after the ramp-down phase as for heat pain, we shortened the stimulus duration for pressure pain by 0.6 s since this was the difference between the average duration for heat (17.76 s) and pressure (17.16) to avoid motor-induced activation. Additionally, the six motion parameters were augmented by their derivatives and the squares of parameters and derivatives resulting in a total of 24 parameters^86^. An additional spike correction was performed, where individual volumes with voxels with a deviation of > 0.6 mm between each volume with its preceding volume of each run were individually modeled. In addition, we included RETROICOR^87^ based physiological noise regressors to account for cardiac and respiratory-related motion^88^. This technique determines cardiac- and respiratory-related noise by allotting cardiac and respiratory phases to individual volumes within a time series. Subsequently, these assigned phases are utilized in a low-order Fourier expansion to generate time-course regressors elucidating signal variations attributed to cardiac or respiratory activities. A refined physiological noise model was applied, computing three cardiac and four respiratory harmonics, along with a multiplicative term incorporating interactions between cardiac and respiratory noise^89^. These 18 regressors were also included in the first-level analyses. The contrast images from the first level analysis were then spatially normalized to MNI space using individual deformation fields (i.e. combining nonlinear coregistration and DARTEL spatial normalization) and finally smoothed using a 6 mm FWHM Gaussian smoothing kernel. In addition to a hemodynamic response function (HRF) model, we also used a Finite Impulse Response model (FIR) to gain a better understanding of the blood-oxygenation level dependent (BOLD) time courses of both noxious stimuli and their intensities. The duration spanned 30.6 s (17 bins of 1.8 s) starting 2 s before stimulus onset. This model was only used to visualise the BOLD time course after the stimulus onset, but was not used for statistical comparisons.

### Second-level analyses

For the second level analysis, the spatially normalised and smoothed first-level contrast images were used in the random effects group-level factorial design. Within that framework, we calculated a conjunction analysis of the contrasts heat > baseline and pressure > baseline to investigate the overlapping activation of both modalities. To identify regions that were distinctly active in heat and pressure pain we calculated the contrasts heat > pressure and pressure > heat, individually. To identify common activation in the stimulus intensity encoding of both modalities, we calculated the conjunction for the parametric contrasts for heat and pressure as well as the interaction between stimulus intensity and modality. For the second model, we calculated the contrasts: interaction of stimulus half (‘early’ and ‘late’ pain) and modality (pressure and heat) with a positive and negative contrast weight across stimulus intensities. Furthermore, previous studies have separated longer pain durations into discrete phases (‘early’ and ‘late’) when investigating the role of endogenous opioids^62–64^ or placebo effects^62,65^. Since we also employed a long stimulus duration, we further estimated contrasts by comparing the ‘early’ and ‘late’ pain phases for each modality individually. To correct for multiple comparisons the brain results are reported at the whole brain level corrected for family-wise-error rate (FWE) and denoted as *p*_*WB-FWE*_ = x.xx. For descriptive purposes, some uncorrected brain results are reported and denoted as *p*_*uncorr*_ = x.xx.

### Neurologic Pain Signature (NPS) analysis

To further investigate compound activity across key brain regions involved in pain perception in both modalities, we employed the NPS. The NPS assigns different weights to voxels depending on their involvement in nociceptive processing^12^. We used the 6 spatially normalised and smoothed first-level contrast images of each intensity contrast (VAS 30, 50, 70) for each modality (pressure and heat) from the first-level GLM and multiplied these with the NPS voxel weights. The resulting NPS scores for each subject and contrast were entered into an LMER model with stimulus intensity, modality, the interaction term, and treatment order as fixed effects and subject as a random effect. Furthermore, we aimed to extract the time course across the NPS for each modality and stimulus intensity. Each of the 17 FIR parameter estimate images for each modality and stimulus intensity was multiplied with the NPS. This resulted in a time course spanning 30.6 s with a start at 2 s before the stimulus onset for heat and pressure pain at each stimulus intensity.

## Supplementary Information

Supplementary Information.

## Data Availability

All data needed to evaluate the conclusions in the paper are present in the paper and/or the Supplemental Materials. The datasets generated during and/or analysed during the current study are available from the corresponding author on reasonable request. The code will be made available after publication on a public repository (GitHub).
