# Supplementary material for: Comparing neural responses to cutaneous heat and pressure pain in healthy participants
Source: Sci Rep. 2025 Apr 24;15:14387. doi: 10.1038/s41598-025-99247-7 (PMC12022288; doi:10.1038/s41598-025-99247-7)
Supplement: Supplementary file 1 — Supplementary Information. [file 41598_2025_99247_MOESM1_ESM.docx]

Supplementary Materials for

**Comparing neural responses to cutaneous heat and pressure pain in healthy participants**

Authors: Janne I. Nold^*1^, Alexandra Tinnermann^1^, Tahmine Fadai^1^, Marilyn Mintah, Marie-Sophie Morgenroth & Christian Büchel^1^

*Corresponding author: Janne Nold

^1^Department of Systems Neuroscience, University Medical Centre Hamburg Eppendorf, Hamburg, Germany

# **Supplemental Materials**

The original study was a randomized controlled study and implemented a within-subject design across three distinct days. Each session was scheduled to occur approximately seven days apart, ensuring a minimum duration of three days between appointments. The first day, designated as the calibration day, transpired outside the MR scanner and was crucial for establishing baseline measurements, including blood pressure, heart rate (HR), and oxygen saturation (SPO2). These measurements were obtained using a blood pressure cuff (boso medicus uno, Bosch+Sohn) and a pulse oximeter (Pulox® Pusloximeter, Novidion GmbH).

An important feature of the original study was its meticulous calibration procedure. This calibration process involved precise individual adjustments of thermal and pressure stimuli, all conducted in a pseudo-randomised order across participants to maintain consistency. To simulate the MR scanner environment, participants remained in a supine position with the blinds lowered during the calibration. Initially, they were exposed to two low stimuli (10/20 kPa or 41°C/42°C) for familiarization purposes. After this initial exposure, six adaptive stimuli were presented to assess the participants' pain thresholds, allowing their responses to be rated on a binary scale (painful vs. not painful). The resulting pain threshold served as a critical reference point for a linear regression algorithm that collected pain ratings across an array of intensities on a Visual Analog Scale (VAS) featuring values of 10, 30, 50, 70, and 90. On the second experimental day, an anatomical scan (T1) was performed alongside a recalibration of thermal and pressure stimuli. This recalibration relied on the intensities established on the calibration day and was adjusted according to ratings provided during the MR scanning process for VAS values of 30, 50, and 70. This recalibration was essential to account for possible differences in sensory perception due to the unique environment of the MR scanner. Moreover, it aimed to acclimate participants to the procedural steps, minimizing transition times between the cycle ergometer and the MR scanner. Participants were required to evaluate its painfulness by responding to the question, “How painful was the last stimulus?” utilizing the VAS, which ranged from 0 (minimally painful) to 100 (almost unbearably painful). Participants were instructed to rate the maximum pain over the 17-second pain. This assessment was conducted using the left and right keys of a button box operated with their right hand. The calibration process concluded once sufficient rating coverage was achieved for all target intensity levels. This calibration intended to identify super-threshold intensities (in °C and kPa) corresponding to specific VAS values (30, 50, and 70), ensuring that comparable stimuli were used across all participants.

Each experimental day was conducted at least three days apart (median = 7 days, *M* = 12.35 days, *SD* = 14.5 days) and was identical in structure, with the only difference being the pharmacological treatment administered (naloxone vs. saline). Each experimental day commenced in the morning, starting at 8:15 a.m. with a maximum variation of four hours between participants, effectively controlling for any potential circadian effects^1^. Blood pressure and SPO2 measurements were consistently monitored before each cycling block. Furthermore, each experimental day consisted of four blocks, each incorporating a ten-minute cycling unit at either a moderate-high intensity (91-106% FTP) or a low intensity (55% FTP). These cycling units were ordered in a pseudo-randomized manner across participants and were immediately followed by an MR scan. Inside the MR scanner, participants received 9 heat and 9 pressure pain stimuli in an alternating fashion at each stimulus intensity level (30, 50, 70). After each stimulus application, participants were required to evaluate its painfulness by responding to the question, “How painful was the last stimulus?” on the VAS, which ranged from 0 (minimally painful) to 100 (almost unbearably painful).

As for the drug intervention, participants were instructed to recline in a supine position before the administration of a bolus dose of naloxone (0.15 mg/kg; Naloxon-ratiopharm® 0.4 mg/ml) or saline (Isotone Kochsalz-Lösung 0.9%, Braun) through peripheral venous access in the right lower arm. This process was handled with care to ensure participant comfort, with two individuals receiving intravenous access in the back of their right hand after confirming verbal consent. Following the bolus administration, an intravenous infusion of naloxone (0.2 mg/kg/h diluted in saline) or saline commenced via an infusion pump (Perfusor® Space, Braun). This infusion was pivotal as it began simultaneously with the first ten-minute cycling block, ensuring that naloxone reached peak blood plasma levels^2^ before the participants were positioned in the MR scanner. Throughout the entirety of the study—including cycling blocks and pain stimulation inside the MR scanner—the intravenous infusion persisted, providing a steady supply of either naloxone or saline. Notably, due to naloxone's relatively short half-life (~70 minutes in blood plasma), this method maintained a stable plasma concentration throughout the study, resulting in effective central opioid blockade^2,3^. The experimenter (J.N.) and student assistants remained blind to the pharmacological interventions, ensuring objectivity, with only the study physician (T. F.) aware of the specific treatments administered to prevent contamination of participants' expectations. Unblinding of the participants occurred post-experiment in a private setting under the guidance of the study physician, thereby preventing any potential expectation induction among the experimenter and student assistants.

# **
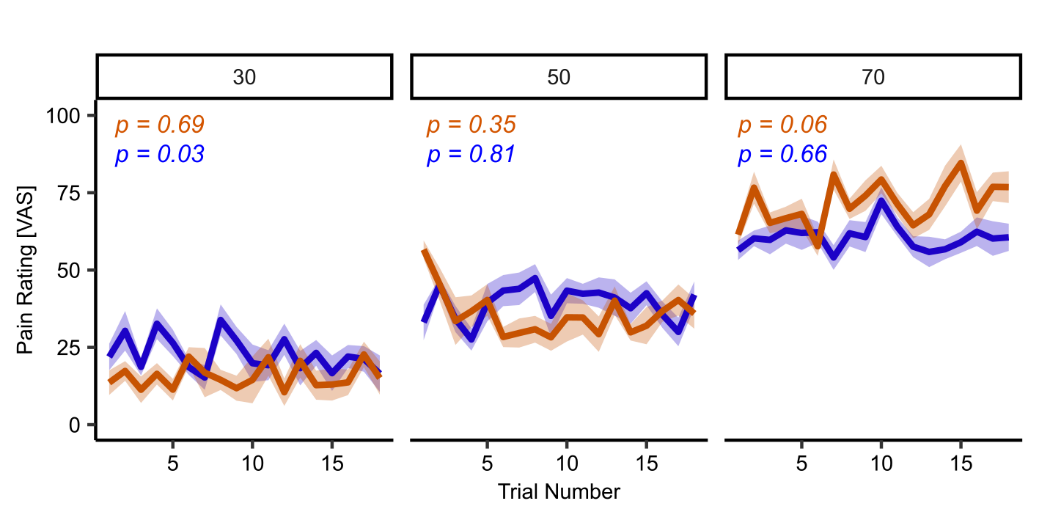
Fig. S1. Mean stimulus ratings across all trials averaged across blocks showed neither sensitisation nor habituation for pressure (blue) or heat (orange) pain.** Lines depict pain ratings across 18 trials per block at each stimulus intensity (left VAS 30; middle VAS 50, right VAS 70). The shaded areas around the curves represent the SEM. P-values depict the significance level of the main effect ‘trial number’ from LMER models (including the subject as a random effect) for each modality and stimulus intensity. Only at pressure VAS 30 a significant effect of trial number was evident. Overall, this suggests no habituation/sensitisation effects for heat or pressure pain across trials. The complete model output can be found in Supplemental Table S9.

# **
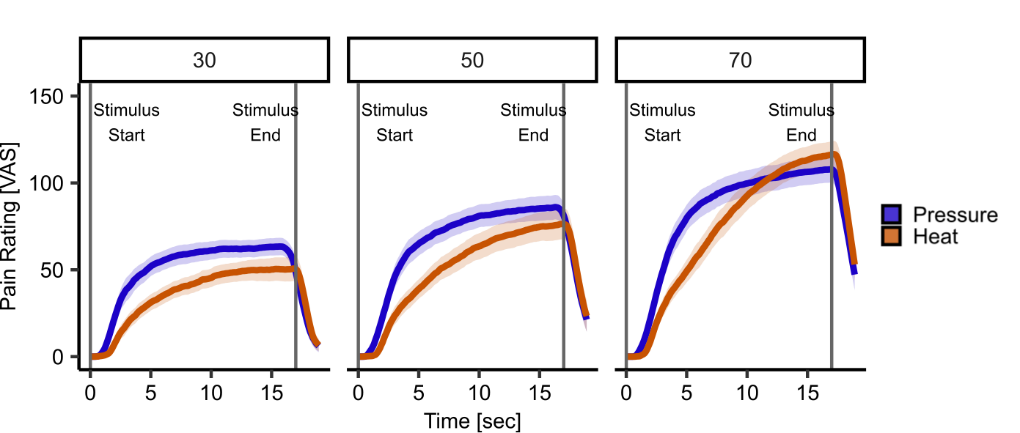
Fig. S2. Behavioural online ratings from follow-up study (*N* = 10, different participants).** Pain ratings are shown at each stimulus intensity [VAS 30 (left panel), VAS50 (middle panel), VAS70 (right panel)] for heat (orange) and pressure (blue) pain. Online ratings were sampled at 70-75 samples/second throughout the stimulus duration (17 seconds) and 2 seconds after the stimulus end. The continuous ratings were interpolated at 0.9 seconds (1/2 of TR 1.8 seconds). AVAS rating of 0 reflected “no perception”, 50 represents the pain threshold (‘minimally painful’), and 150 the pain tolerance (‘almost unbearably painful’). Participants saw the verbal but not numerical anchor points of the VAS scale. Paired samples t-tests between the ratings averaged across the first and second half of the stimulus duration between both modalities at each stimulus intensity were conducted. When averaging the ratings across the first stimulus half (0-8.5s), there is a significant difference between the modalities at all stimulus intensities (VAS 30: *t*(119) = -13.16, *p* < 2.2e-16; VAS 50: *t*(119) = -13.27, *p* < 2.2e-16; VAS 70: *t*(119) = -10.50, *p* < 2.2e-16). Averaging the ratings across the first stimulus half (8.5s-17s), a significant difference was evident at VAS 30 (*t*(119) = -6.94, *p* = 2.195e-10)) and VAS 50 (*t*(119) = -5.13, *p* = 1.159e-06) but not at VAS 70 (*t*(119) = 0.30, *p* = 0.77). The shaded areas around the curves represent the SEM. The grey solid lines indicate the stimulus start and end.

# **
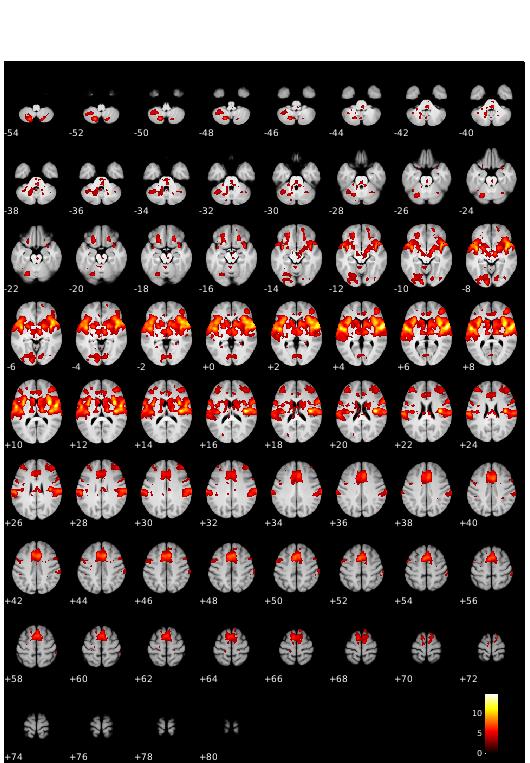
Fig. S3. Uncorrected activation map for main effect heat**. BOLD activation at *p_uncorr_* < 0.001 uncorrected superimposed onto mean T1 along 134 slices (2 slice steps) in axial view.

# **
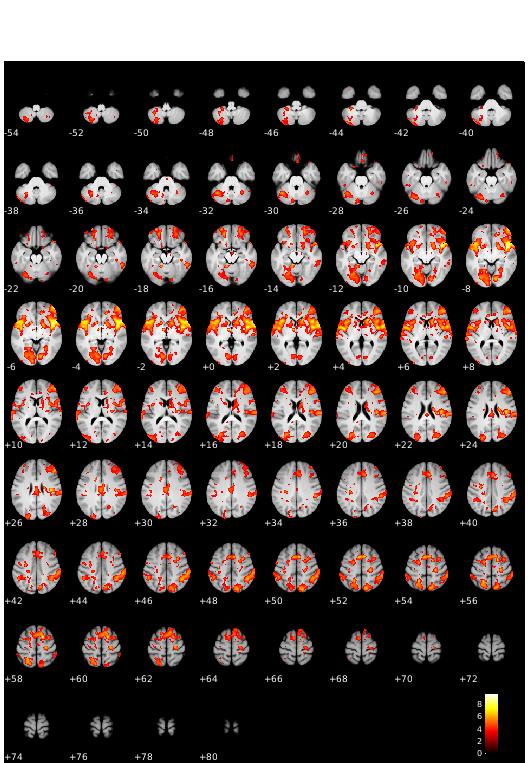
Fig. S4. Uncorrected activation map for main effect pressure**. BOLD activation at *p_uncorr_* < 0.001 uncorrected superimposed onto mean T1 along 134 slices (2 slice steps) in axial view.


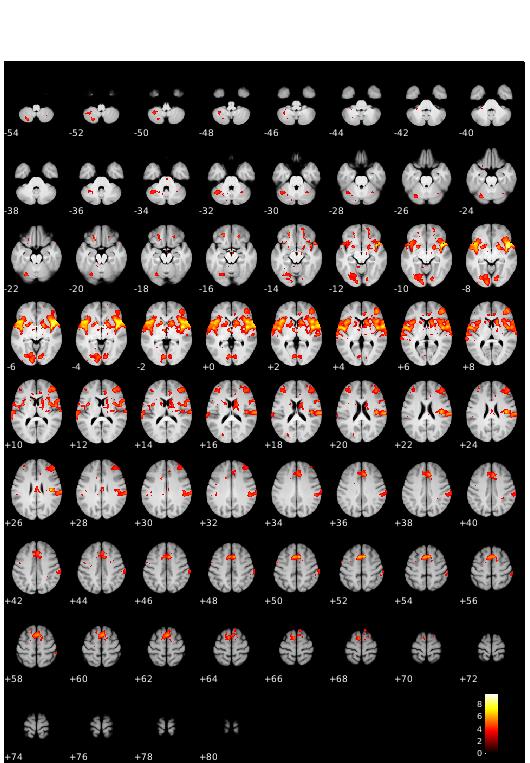


# **Fig. S5. Uncorrected activation map conjunction analysis of heat and pressure pain**. BOLD activation at *p_uncorr_* < 0.001 uncorrected superimposed onto mean T1 along 134 slices (2 slice steps) in axial view.


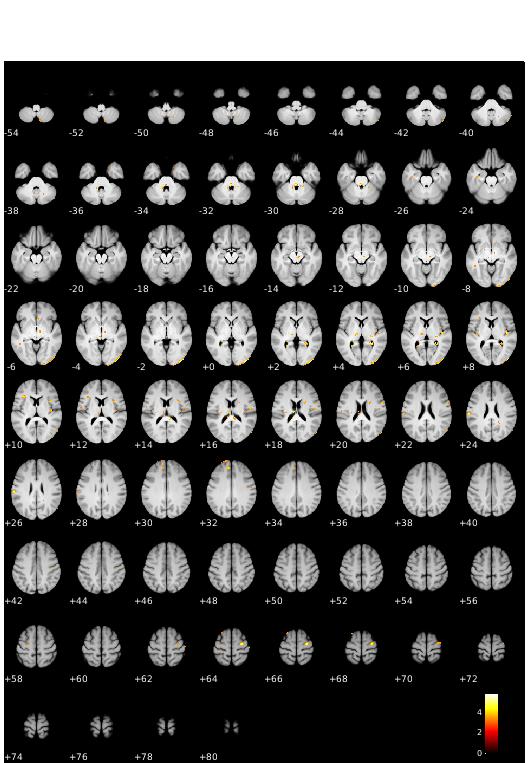


# **Fig. S6. Uncorrected activation map for contrast heat > pressure.** BOLD activation at *p_uncorr_* < 0.001 uncorrected superimposed onto mean T1 along 134 slices (2 slice steps) in axial view.


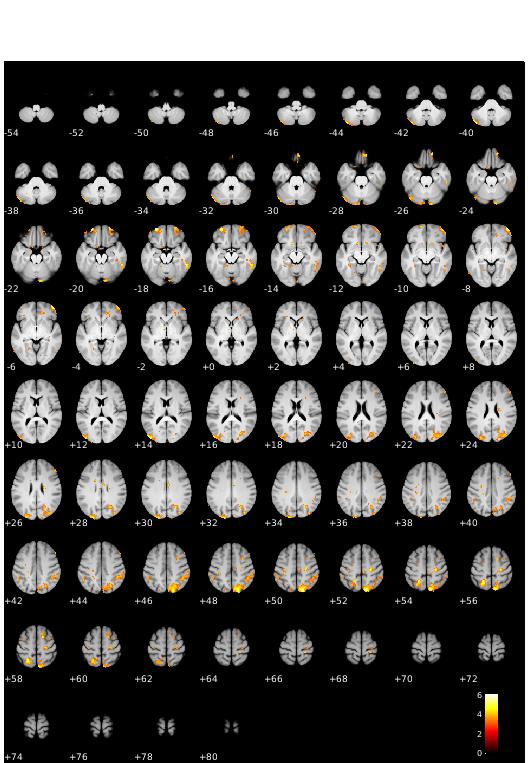


# **Fig. S7. Uncorrected activation map for contrast pressure > heat.** BOLD activation at *p_uncorr_* < 0.001 uncorrected superimposed onto mean T1 along 134 slices (2 slice steps) in axial view.


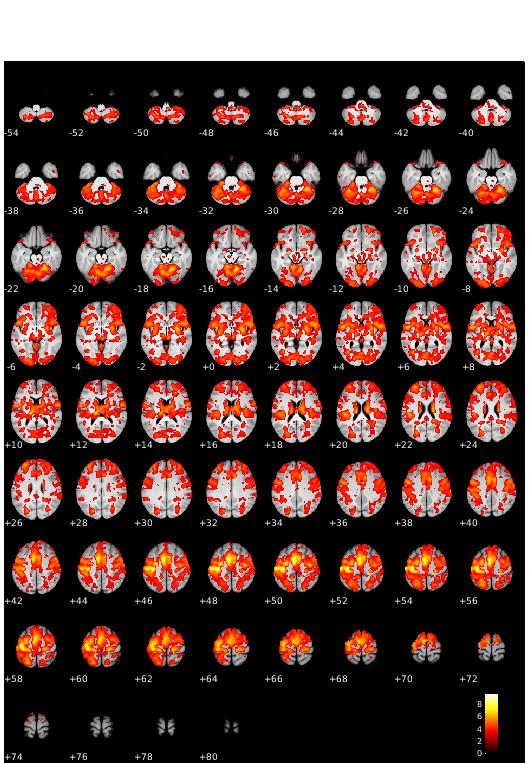


# **Fig. S8. Uncorrected activation map for contrast heat late > early pain.** BOLD activation at *p_uncorr_* < 0.001 uncorrected superimposed onto mean T1 along 134 slices (2 slice steps) in axial view.


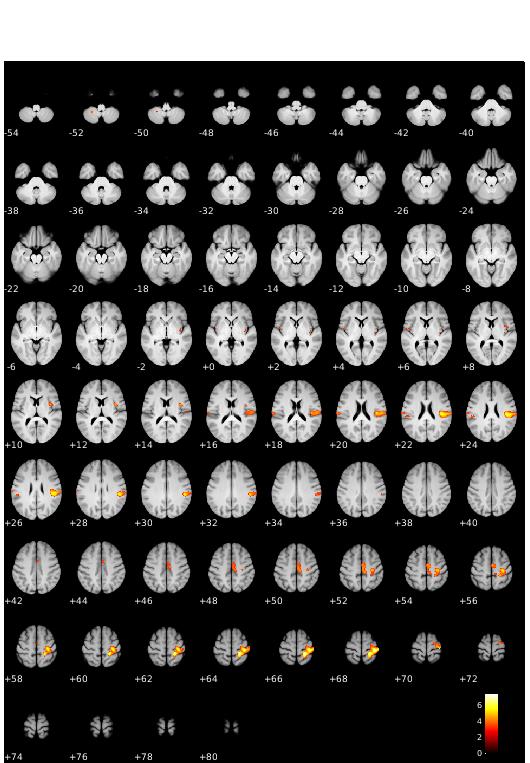


# **Fig. S9. Uncorrected activation map for contrast pressure early > late pain.** BOLD activation at *p_uncorr_* < 0.001 uncorrected superimposed onto mean T1 along 134 slices (2 slice steps) in axial view.


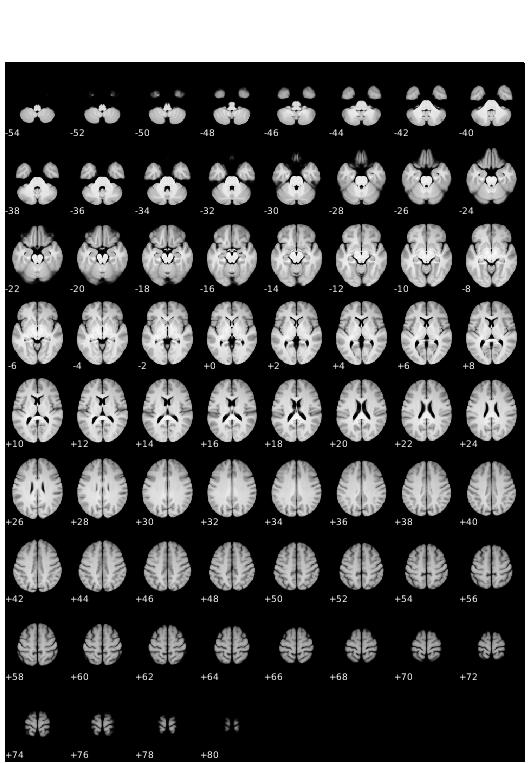


# **Fig. S10. Uncorrected activation map for contrast heat early > late pain.** BOLD activation at *p_uncorr_* < 0.001 uncorrected superimposed onto mean T1 along 134 slices (2 slice steps) in axial view.


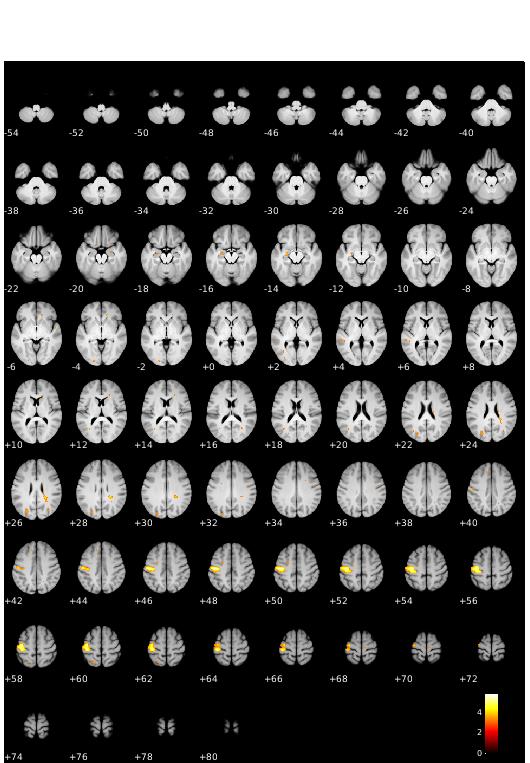


# **Fig. S11. Uncorrected activation map for contrast pressure late > early pain.** BOLD activation at *p_uncorr_* < 0.001 uncorrected superimposed onto mean T1 along 134 slices (2 slice steps) in axial view.

# **
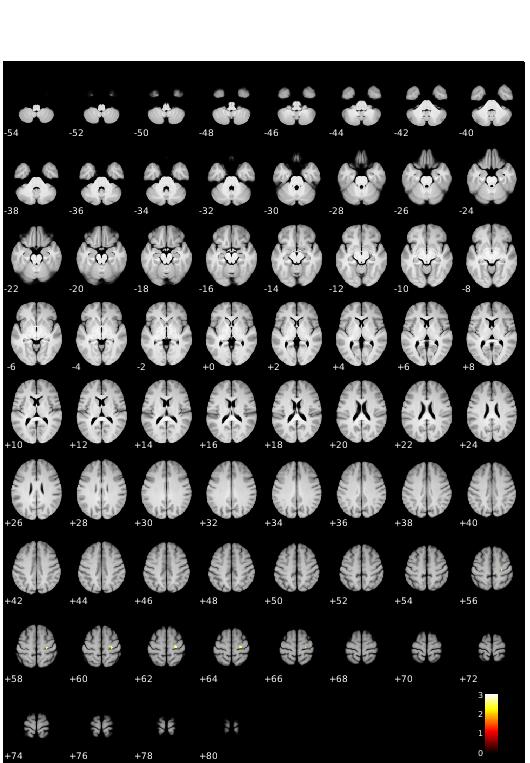
Fig. S12. Uncorrected activation map conjunction analysis of parametric heat (70>50>30) and parametric pressure (70>50>30) pain**. BOLD activation at ***p_uncorr_* < 0.01** uncorrected superimposed onto mean T1 along 134 slices (2 slice steps) in axial view.

# **
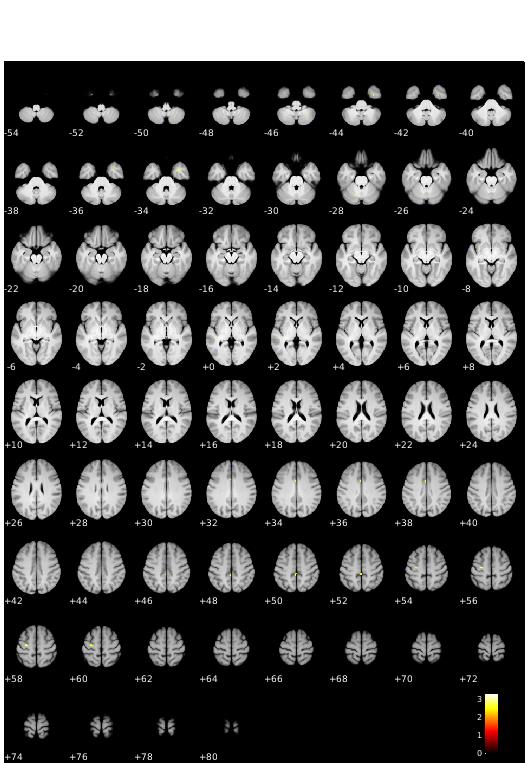
Fig. S13. Uncorrected activation map for the interaction of modality and stimulus intensity**. BOLD activation at ***p_uncorr_* < 0.01** uncorrected superimposed onto mean T1 along 134 slices (2 slice steps) in axial view.

# **
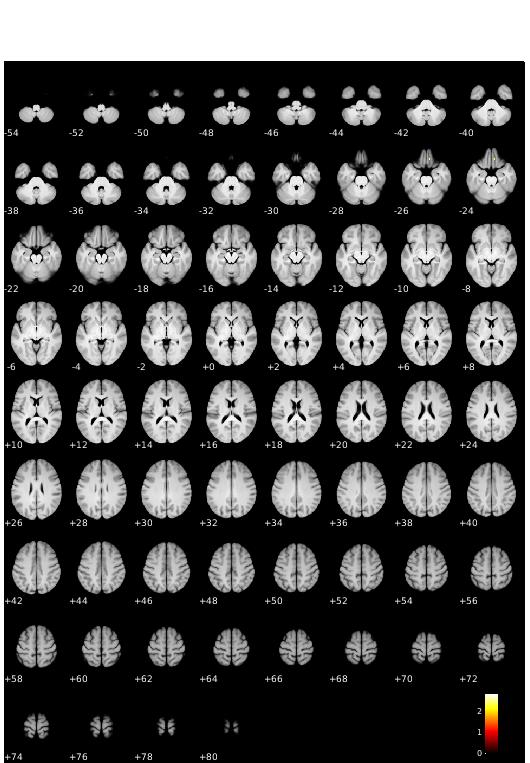
Fig. S14. Uncorrected activation map for the negative interaction of modality and stimulus intensity**. BOLD activation at ***p_uncorr_* < 0.01** uncorrected superimposed onto mean T1 along 134 slices (2 slice steps) in axial view.


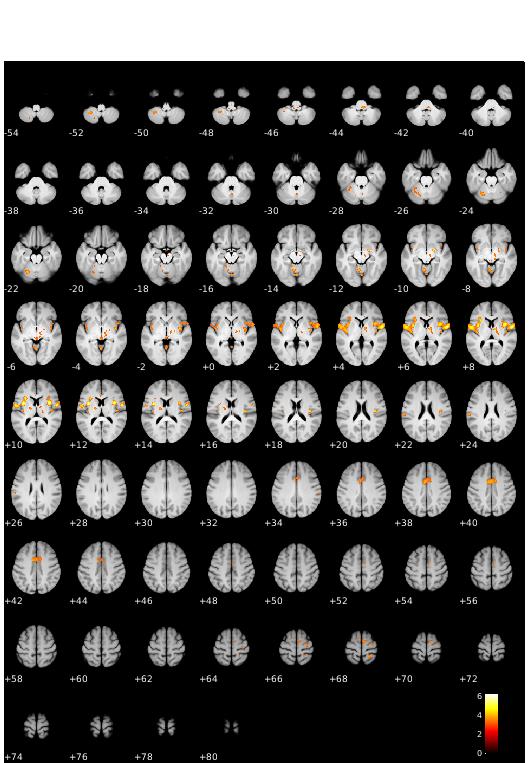


# **Fig. S15. Uncorrected activation map for contrast parametric heat (VAS 70 > VAS 50 > VAS 30).** BOLD activation at *p_uncorr_* < 0.001 uncorrected superimposed onto mean T1 along 134 slices (2 slice steps) in axial view.

#
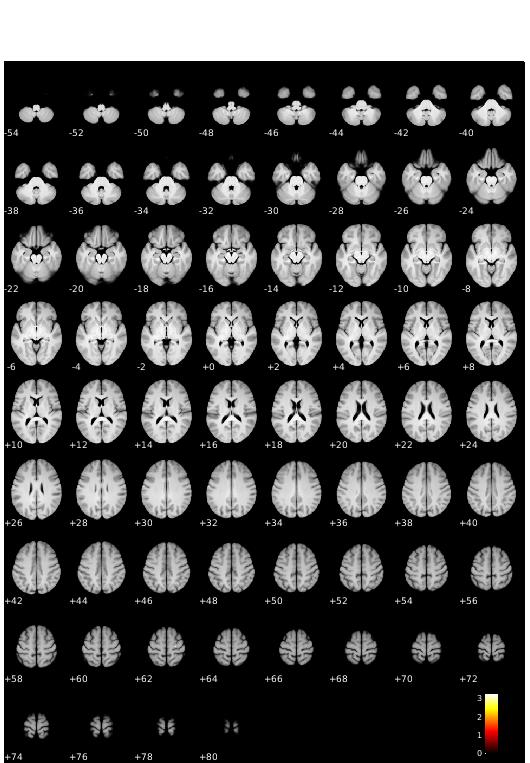
**Fig. S16. Uncorrected activation map for contrast parametric pressure (VAS 70>VAS50> VAS30).** BOLD activation at *p_uncorr_* < 0.001 uncorrected superimposed onto mean T1 along 134 slices (2 slice steps) in axial view.

# **
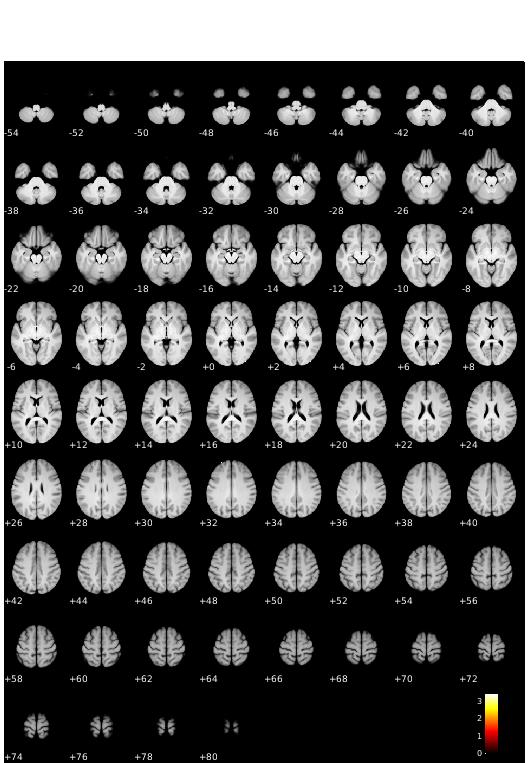
Fig. S17. Uncorrected activation map for contrast Heat 30 > Pressure 30**. BOLD activation at *p_uncorr_* < 0.001 uncorrected superimposed onto mean T1 along 134 slices (2 slice steps) in axial view.

# **
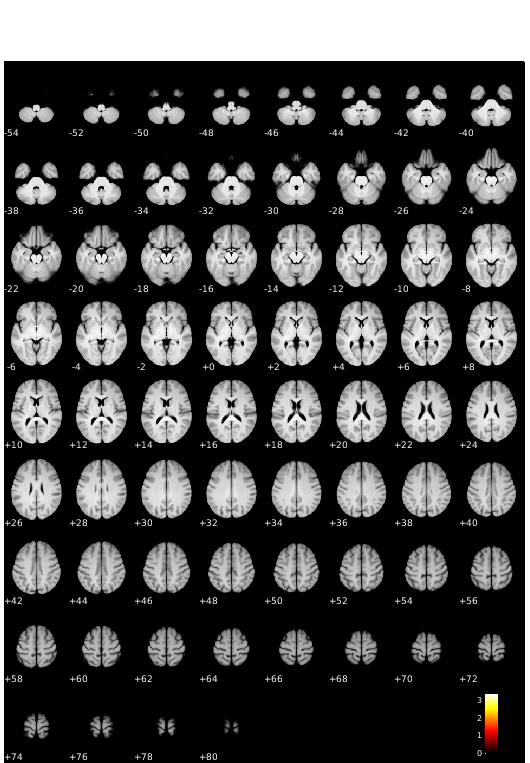
Fig. S18. Uncorrected activation map for contrast Heat 50 > Pressure 50**. BOLD activation at *p_uncorr_* < 0.001 uncorrected superimposed onto mean T1 along 134 slices (2 slice steps) in axial view.

# **
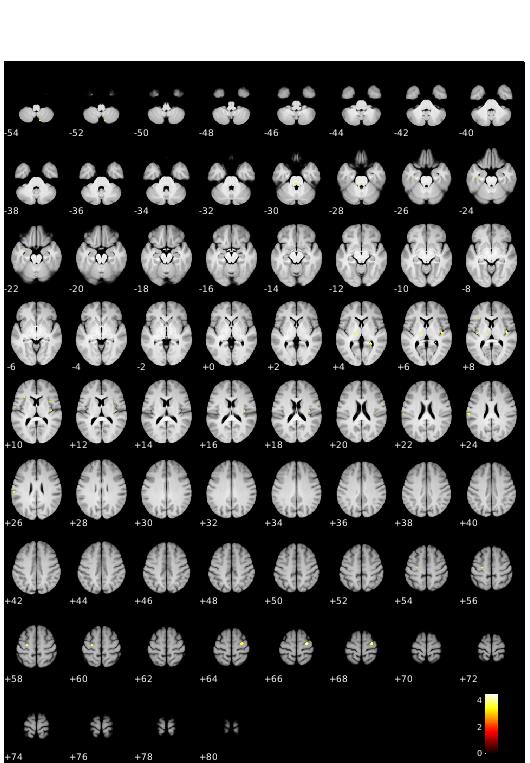
Fig. S19. Uncorrected activation map for contrast Heat 70 > Pressure 70**. BOLD activation at *p_uncorr_* < 0.001 uncorrected superimposed onto mean T1 along 134 slices (2 slice steps) in axial view.

# **
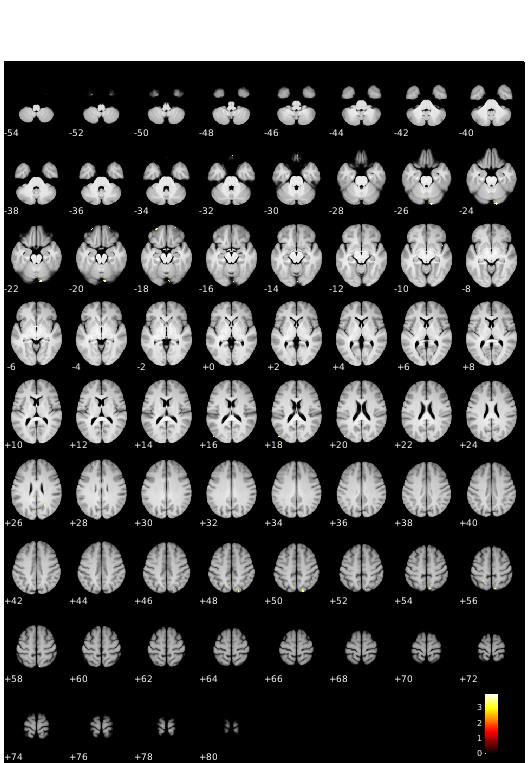
Fig. S20. Uncorrected activation map for contrast Pressure 30 > Heat 30**. BOLD activation at *p_uncorr_* < 0.001 uncorrected superimposed onto mean T1 along 134 slices (2 slice steps) in axial view.

# **
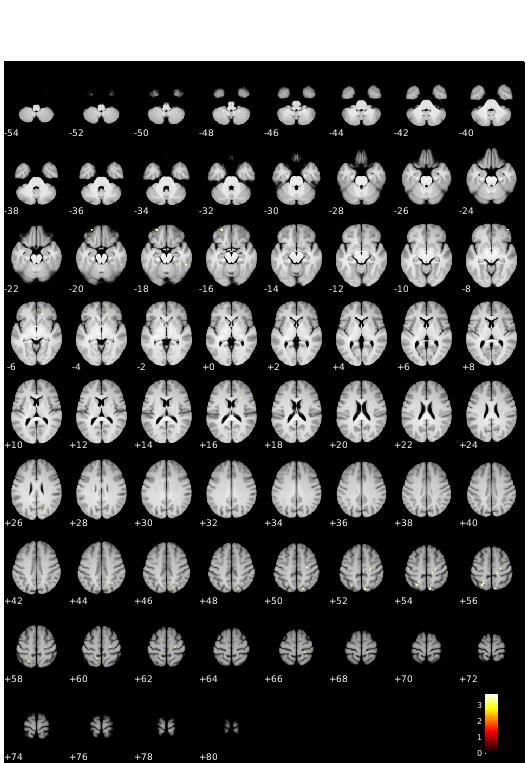
Fig. S21. Uncorrected activation map for contrast Pressure 50 > Heat 50**. BOLD activation at *p_uncorr_* < 0.001 uncorrected superimposed onto mean T1 along 134 slices (2 slice steps) in axial view.

# **
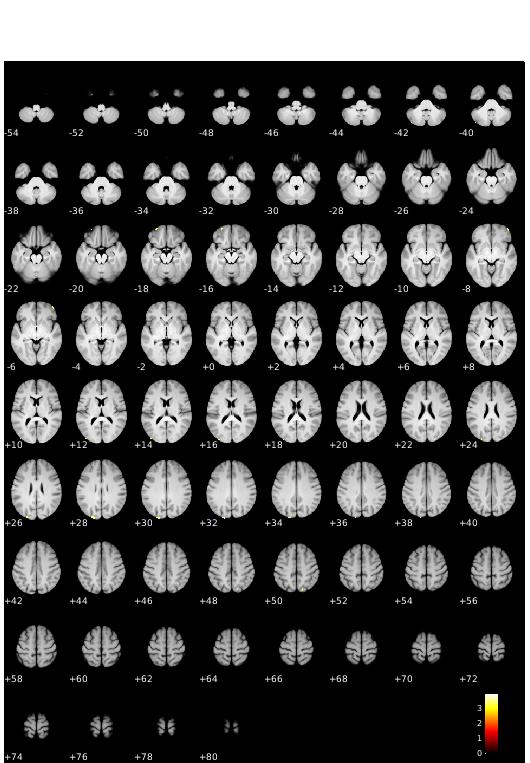
Fig. S22. Uncorrected activation map for contrast Pressure 70 > Heat 70**. BOLD activation at *p_uncorr_* < 0.001 uncorrected superimposed onto mean T1 along 134 slices (2 slice steps) in axial view.

# **
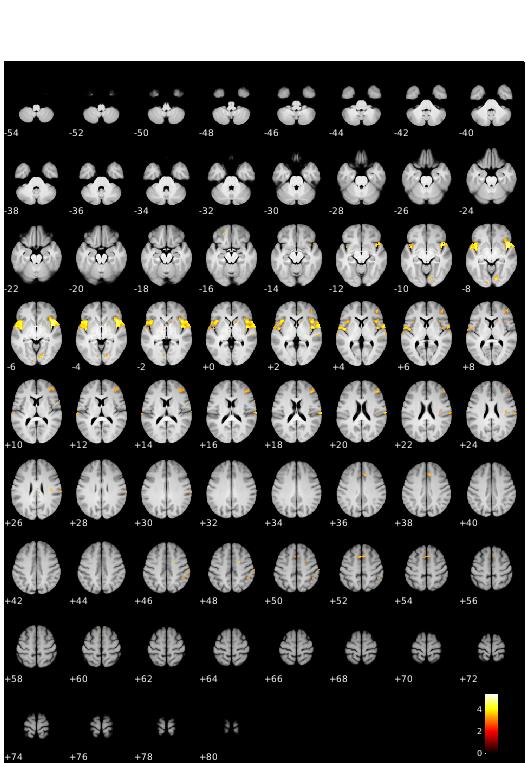
Fig. S23. Uncorrected activation map for conjunction Pressure 30 and Heat 30**. BOLD activation at *p_uncorr_* < 0.001 uncorrected superimposed onto mean T1 along 134 slices (2 slice steps) in axial view.

# **
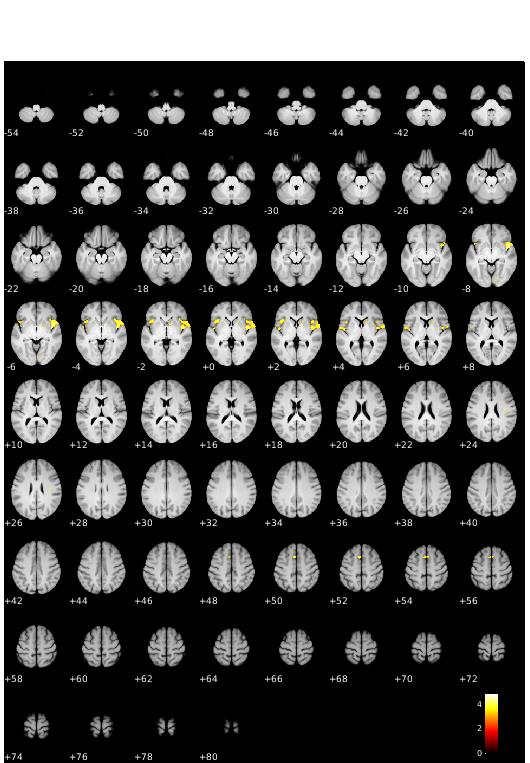
Fig. S24. Uncorrected activation map for conjunction Pressure 50 and Heat 50**. BOLD activation at *p_uncorr_* < 0.001 uncorrected superimposed onto mean T1 along 134 slices (2 slice steps) in axial view.

# **
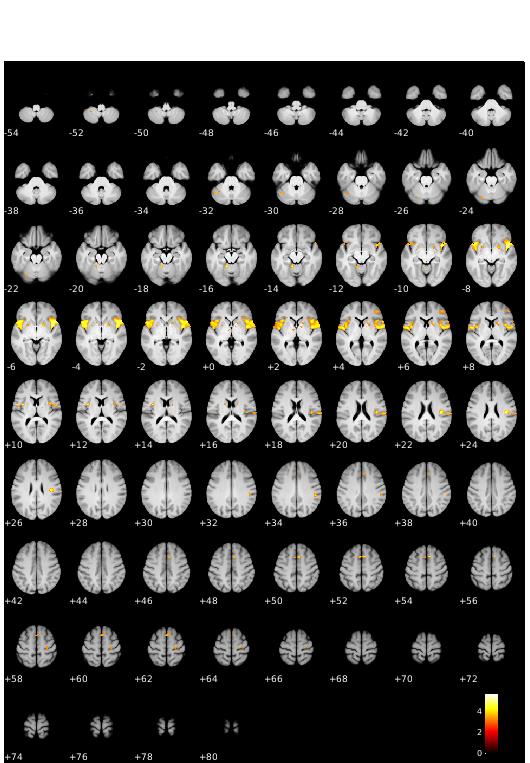
Fig. S25. Uncorrected activation map for conjunction Pressure 70 and Heat 70**. BOLD activation at *p_uncorr_* < 0.001 uncorrected superimposed onto mean T1 along 134 slices (2 slice steps) in axial view.

# **Table S1. LMER model output for modality on pain ratings.**

| Fixed Effects | Estimate | *SE* | *df* | *t* | *p* |
| --- | --- | --- | --- | --- | --- |
| Intercept | 38.01 | 2.35 | 44.75 | 16.21 | <2×10^-16^ |
| Modality | -0.39 | 1.38 | 1346.56 | -0.29 | 0.78 |
| Treatment_order | 7.18 | 3.36 | 37 | 2.13 | 0.04 |
| LMER = linear mixed effects model, *SE* = standard error, *df* = degrees of freedom. Subject and trial number were included as random effects. | | | | | |

# **Table S2. LMER model output for stimulus intensity on heat pain ratings.**

| Fixed Effects | Estimate | *SE* | *df* | *t* | *p* |
| --- | --- | --- | --- | --- | --- |
| Intercept | -32.50 | 3.13 | 97.50 | -10.37 | <2×10^-16^ |
| Stimulus Intensity | 1.39 | 0.04 | 660.32 | 35.72 | <2×10^-16^ |
| Treatment_order | 8.68 | 3.66 | 37 | 2.37 | 0.02 |
| LMER = linear mixed effects model, *SE* = standard error, *df* = degrees of freedom. Subject and trial number were included as random effects. | | | | | |

# **Table S3. LMER model output for stimulus intensity on pressure pain ratings.**

| Fixed Effects | Estimate | *SE* | *df* | *t* | *p* |
| --- | --- | --- | --- | --- | --- |
| Intercept | -7.93 | 3.27 | 71.63 | -2.42 | 0.02 |
| Stimulus Intensity | 0.93 | 0.04 | 660.69 | 26.62 | <2×10^-16^ |
| Treatment_order | 5.63 | 4.18 | 37.01 | 1.35 | 0.19 |
| LMER = linear mixed effects model, *SE* = standard error, *df* = degrees of freedom. Subject and trial number were included as random effects. | | | | | |

# **Table S4. Stimulus intensities to evoke VAS ratings at 30, 50, and 70 for heat (temperature in °C) and pressure (in kPa) stimuli.**

| VAS | Mean (in °C) | SD (in °C) | SE (in °C) | Mean (in kPa) | SD (in kPa) | SE (in kPA) |
| --- | --- | --- | --- | --- | --- | --- |
| 30 | 44.7 | 1.05 | 0.54 | 35.25 | 14.70 | 0.52 |
| 50 | 45.7 | 0.90 | 0.54 | 49.41 | 16.40 | 0.59 |
| 70 | 46.7 | 0.83 | 0.55 | 62.79 | 17.20 | 0.68 |
| VAS = Visual Analog Scale; kPa = kilopascal; SD = standard deviation; SE = standard error. | | | | | | |

# **Table S5. Paired samples t-test between intensity levels for heat and pressure pain.**

| Contrast | T | *df* | *p* |
| --- | --- | --- | --- |
| P 30 – P 50 | -13.74 | 233 | < 0.001 |
| P 50 – P 70 | -17.09 | 232 | < 0.001 |
| P 30 – P 70 | -28.60 | 232 | < 0.001 |
| H 30 – H 50 | -15.51 | 232 | < 0.001 |
| H 50 – H 70 | -25.30 | 232 | < 0.001 |
| H 30 – H 70 | -34.99 | 233 | < 0.001 |
| P = pressure, H = heat; 30, 50, 70 = Stimulus intensity at VAS 30, 50, and 70. T = test statistic, df = degrees of freedom. | | | |

# **Table S6. LMER model output for interaction modality and stimulus intensity on pain ratings.**

| Fixed Effects | Estimate | *SE* | *df* | *t* | *p* |
| --- | --- | --- | --- | --- | --- |
| Intercept | -8.62 | 3.01 | 121.68 | -2.87 | 0.005 |
| Stimulus Intensity | 0.93 | 0.04 | 1359.99 | 23.74 | <2×10^-16^ |
| Modality | -23.11 | 2.92 | 1359.28 | -7.91 | 5.35×10^-15^ |
| Treatment_order | 7.15 | 3.36 | 37 | 2.13 | 0.04 |
| Stimulus Intensity×Modality | 0.45 | 0.06 | 1359.75 | 8.17 | 7.25×10^-16^ |
| LMER = linear mixed effects model, *SE* = standard error, *df* = degrees of freedom. Subject and trial number were included as random effects. | | | | | |

# **Table S7. LMER model output for main effect stimulus intensity on difference pain ratings [Heat – Pressure].**

| Fixed Effects | Estimate | *SE* | *df* | *t* | *p* |
| --- | --- | --- | --- | --- | --- |
| Intercept | -24.45 | 4.25 | 111.18 | -5.75 | 7.91×10^-8^ |
| Stimulus Intensity | 0.45 | 0.07 | 77 | 6.87 | 1.44×10^-9^ |
| Treatment_order | 3.09 | 4.07 | 37 | 0.76 | 0.45 |
| LMER = linear mixed effects model, *SE* = standard error, *df* = degrees of freedom. Subject and trial number were included as random effects. | | | | | |

# **Table S8. Paired samples t-tests between modalities (heat vs. pressure) at each intensity level.**

| Contrast | T | *df* | *p* |
| --- | --- | --- | --- |
| P 30 – H 30 | 5.79 | 233 | < 0.001 |
| P 50 – H 50 | 2.65 | 232 | 0.008 |
| P 70 – H 70 | -6.29 | 232 | < 0.001 |
| P = pressure, H = heat; 30, 50, 70 = Stimulus intensity at VAS 30, 50, and 70. T = test statistic, df = degrees of freedom | | | |

# **Table S9**. **The main effect of trial number in individual LMER models for heat and pressure pain at each stimulus intensity.**

|  | Estimate | *SE* | *df* | *t* | *p* |
| --- | --- | --- | --- | --- | --- |
| Heat VAS 30 | -0.06 | 0.15 | 197.30 | -0.40 | 0.69 |
| Heat VAS 50 | -0.20 | 0.22 | 203.32 | -0.94 | 0.35 |
| Heat VAS 70 | 0.39 | 0.20 | 196.04 | 1.92 | 0.06 |
| Pressure VAS 30 | -0.39 | 0.18 | 196.72 | -2.15 | 0.03 |
| Pressure VAS 50 | -0.05 | 0.19 | 196.94 | -0.24 | 0.81 |
| Pressure VAS 70 | 0.09 | 0.19 | 195.39 | 0.45 | 0.66 |
| 30, 50, 70 = Stimulus intensity at VAS 30, 50, and 70; LMER = linear mixed effects model, *SE* = standard error, *df* = degrees of freedom. The subject was included as a random effect. | | | | | |

|  | Overall Mean (SD) | Females Mean (SD) | Males Mean (SD) |
| --- | --- | --- | --- |
| *N* | 10 | 7 | 3 |
| Age (years) | 27.7 (6.25) | 28.57 (7.23) | 25.67 (3.21) |
| Weight (kg) | 72.1 (13.46) | 66.57 (6.68) | 85 (18.03) |
| Height (cm) | 174.5 (9.12) | 170.71 (7.04) | 183.33 (7.64) |
| BMI (kg/(cm/100)²) | 23.60 (3.28) | 22.95 (3.03) | 25.12 (4.00) |
| SD = Standard deviation. Kg = kilogram. cm = centimetre. | | | |

# **Table S10.** **Participant characteristics of the follow-up sample (*N* = 10).**

# **Table S11. LMER model output for interaction modality and stimulus intensity on Neurologic Pain Signature (NPS) scores.**

| Fixed Effects | Estimate | *SE* | *df* | *t* | *p* |
| --- | --- | --- | --- | --- | --- |
| Intercept | 1.92 | 1.83 | 196.90 | 1.05 | 0.29 |
| Stimulus Intensity | 0.04 | 0.03 | 192 | 1.36 | 0.17 |
| Modality | -3.62 | 2.20 | 192 | -1.65 | 0.10 |
| Treatment_order | -0.32 | 1.54 | 37 | -0.21 | 0.84 |
| Stimulus Intensity×Modality | 0.09 | 0.04 | 192 | 2.27 | 0.02 |
| LMER = linear mixed effects model, *SE* = standard error, *df* = degrees of freedom. The subject was included as a random effect. | | | | | |

# **Table S12. Participant characteristics (*N* = 39).**

|  | Overall Mean (SD) | Females Mean (SD) | Males Mean (SD) |
| --- | --- | --- | --- |
| *N* | 39 | 21 | 18 |
| Age (years) | 26.03 (4.83) | 25.33 (5.10) | 26.83 (4.35) |
| Weight (kg) | 70.95 (12.14) | 63.33 (7.53) | 79.83 (10.38) |
| Height (cm) | 177.10 (9.08) | 170.52 (6.35) | 184.78 (4.58) |
| SD = Standard deviation. Kg = kilogram. cm = centimetre. | | | |
